# Chemotherapy-induced cardiotoxicity in cancer patients: an observational study at an Oncology reference hospital from southeastern Brazil*

**DOI:** 10.1590/1518-8345.7472.4774

**Published:** 2026-01-19

**Authors:** Karoline Neumann, Karolini Zuqui Nunes, Wesley Rocha Grippa, Naira Santos D’Agostini, Sara Isabel Pimentel de Carvalho Schuab, Luís Carlos Lopes-Júnior

**Affiliations:** 1 Universidade Federal do Espírito Santo, Vitória, ES, Brazil. Universidade Federal do Espírito Santo ES Vitória Brazil; 2 Scholarship holder at the Fundação de Amparo à Pesquisa e Inovação do Espírito Santo (FAPES), Brazil. Scholarship holder at the Fundação de Amparo à Pesquisa e Inovação do Espírito Santo Brazil; 3 Universidade Federal do Espírito Santo, Departamento de Enfermagem, Vitória, ES, Brazil. Universidade Federal do Espírito Santo Departamento de Enfermagem ES Vitória Brazil; 4 Scholarship holder at the Conselho Nacional de Desenvolvimento Científico e Tecnológico (CNPq), Brazil. Scholarship holder at the Conselho Nacional de Desenvolvimento Científico e Tecnológico Brazil

**Keywords:** Neoplasms, Antineoplastic Agents, Heart Disease Risk Factors, Biomarkers, Electrocardiogram, Cardiotoxicity

## Abstract

to assess the clinical, inflammatory and electrocardiographic profile of cancer patients undergoing the Pre-chemotherapy phase, with the purpose of early identifying cardiovascular toxicity indicative signs.

an observational and cross-sectional study, conducted between November2022 and December2023 with adult cancer patients in stagesI toIII treated at a hospital from southern Brazil. Sociodemographic, clinical and tumor-related data were collected, in addition to laboratory and electrocardiographic tests performed before the first chemotherapy infusion. Descriptive analyses were carried out in the Rsoftware.

the participants were 84 patients, mostly women (72.6%) with breast cancer diagnoses(57.1%). Prevalence of systemic inflammation was verified, with high C-reactive protein in 35.7% and increased neutrophil-lymphocyte and platelet-lymphocyte ratios in 23.5% and 27.2%, respectively. A significant association was observed between high Body Mass Index and T-wave alterations (p=0.005). Although 10 patients presented prolonged corrected QT intervals, no other statistically significant associations were identified.

the pre-chemotherapy phase revealed relevant inflammatory and electrocardiographic alterations, even in the absence of installed cardiotoxicity. The findings reinforce the importance of baseline cardiovascular evaluations and of multi-professional follow-up in Cardio-Oncology, especially for patients with modifiable risk factors. Longitudinal studies are recommended to elucidate the clinical and prognostic evolution of these alterations.

## Highlights

**(1)** Patients on anthracycline and taxane regimes are at a higher risk of cardiotoxicity. **(2)** C-reactive protein was higher at the first chemotherapy infusion. **(3)** High NLRs and PLRs in up to 27% of the patients before treatment initiation. **(4)** Early risk stratification enables cardiotoxicity management. **(5)** Using biomarkers expands the Precision Nursing potential in individualized care.

## Introduction

At the global level, non-communicable diseases(NCDs) emerge as the main cause of morbidity and mortality in the population. This fact is driven by the demographic transition that contributes to the population aging process, associated with the epidemiological transition characterized by changes in the patterns regarding diseases and mortality; therefore, it is considered an important challenge at the global level^(1)^. Among the NCDs, the group of chronic non-communicable diseases(CNCDs) stand out due to worldwide mortality, with cardiovascular diseases(CVDs) as the main causes of death, responsible for almost 18million cases^(2)^ and followed by neoplasms, which account for almost 10million deaths. In most countries, cancer is the first or second leading cause of death among individuals aged less than 70years old^(3)^.

Significant advances have been made in cancer treatments during the last few years, with new drugs and therapies discovered, thus improving prognoses in cancer patients^(4-5)^. However, despite the benefits contributed by anti-neoplastic therapies, cardiovascular diseases emerged as one of their main adverse effects, contributing to the patients’ morbidity and mortality^(4-6)^. Although chemotherapy agents play an important role for these patients’ outcomes, using some of their classes resulted in an increasing number of side effects and cardiovascular complications, with chemotherapy-induced cardiotoxicity as the most common instance, understood as developing after the treatment^(6)^.

The European Society of Cardiology(ESC) defines cardiovascular toxicity(CVT) as a cardiac lesion that is both functional and structural and is conditioned by cancer treatments(chemotherapy, radiotherapy or even cancer itself)^(7-8)^. In addition, CVT can be divided into acute, sub-acute or late, where its signs and symptoms can emerge some years after the cancer patients’ treatments, affecting cardiac structures and triggering heart failure(HF), coronary arterial disease, cardiac valve disease, arrhythmias, cardiac conduction disease and pericardial disease^(8)^.

At the beginning, cardiotoxicity meant a reduction in the ejection fraction; however, Brazilian Cardio-Oncology GuidelineI from the Brazilian Society of Cardiology redefined such condition as follows: cardiomyopathy with reduced left ventricular ejection fraction(LVEF); HF symptoms; HF-conditioned signs; LVEF reduced from 5% to less than 55% when compared to the baseline value and presenting HF signs or symptoms; or LVEF reduced from 10% to less than 55% without simultaneous signs or symptoms^(9-10)^.

The onset of cardiac toxicity during cancer therapies can limit the treatments, leading to their discontinuation or cessation and potentially worsening the patients’ clinical outcomes^(11)^. Monitoring cardiac diseases and risk factors is fundamental and can improve the patients’ prognoses^(7)^.

Despite recent progress in understanding cardiotoxicity induced by anti-neoplastic agents, most of the studies are focused on late clinical outcomes or on population groups already subjected to multiple treatment cycles, leaving an important gap about cancer patients’ baseline cardiovascular profile before chemotherapy initiation. As pointed out in a systematic review^(12)^, despite the extensive documentation about cardiac toxicity in patients with hematological cancer, there is scarcity of studies researching early inflammatory and electrocardiographic alterations in population groups with solid neoplasms, especially in Latin American contexts and in public services that are a reference in Oncology.

In this sense, our study differentiates itself for unprecedentedly assessing inflammatory markers and electrocardiographic alterations in the Pre-chemotherapy phase among cancer patients in initial to intermediate stages (from I to III), contributing to early detection of possible cardiotoxicity signs even before accumulated exposure to anti-neoplastic therapies. Therefore, this is an original study that seeks to expand understanding about the baseline phase of Cardio-Oncology treatments, with a potential impact on risk stratification and on multi-professional care planning. In this context, the paper aimed at assessing the clinical, inflammatory and electrocardiographic profile of cancer patients in the Pre-chemotherapy phase, with the purpose of early identifying cardiovascular toxicity indicativesigns.

## Method

### Study design

This was an observational and cross-sectional study conducted according to the STROBE (Strengthening the Reporting of Observational Studies in Epidemiology) guidelines^(13)^ to ensure transparency and quality in presenting the data and the results.

### Setting

This research was conducted between November2022 and December2023 at *Afecc-Hospital Santa Rita de Cássia*(HSRC), which is the only High-Complexity Oncology Center (*Centro de Alta Complexidade em Oncologia*,CACON) in Espírito Santo, Brazil, and a state reference in cancer treatments for the entire state, southern Bahia, eastern Minas Gerais and northern Rio de Janeiro^(14)^.

### Participants

Individuals from both genders were selected, aged above 18years old, with anatomopathological diagnoses of malignant neoplasms in stages I, II and III of the disease regardless of the type of tumor, and only new cases of patients undergoing outpatient chemotherapy. The subjects excluded were those in stageIV of the disease, in addition to those under exclusive Palliative Care and those with more than one primary tumor.

### Variables

The sociodemographic data and tumor characterization were exclusively obtained through medical records and by applying a semi-structured questionnaire. Blood was collected to assess biomarkers: hs-CRP and TroponinI (outcome variables). The biomarkers were analyzed in the Tomasi Laboratory, located in *Afecc*-HSRC. It is noted that hemograms were a routine in the hospital and that all the information was therefore extracted directly from the patients’ medical records. For blood collection, each participant provided a peripheral venous blood sample (4ml), collected through venipuncture in the cubital fossa. Vacutainer^®^ ethyldiamine tetra-acetic acid (EDTA) vacuum tubes[Becton Dickinson(BD), Franklin Lakes, NJ,USA] were used to such end. In order to obtain the plasma, the biological samples collected were processed following this protocol: centrifuging the Vacutainer tubes containing blood for 10minutes at 4°C, 581.2g/2,000RPM(Eppendorf Centrifuge5810R). Subsequently, it was all divided into aliquots with a pipette and the plasma, to then be transferred to storage microtubes. All the material divided into aliquots was stored in a freezer at -80C until the biomarker quantification stage.

The cutoff points adopted for the biomarkers were defined based on clinical guidelines and on the current scientific literature. For high-sensitivity C-reactive protein(hs-CRP), the American Heart Association(AHA) classification was used, which categorizes cardiovascular risk as follows: low(<1mg/dL), moderate(1–3mg/dL) and high(>3mg/dL), with due support found in clinical and laboratory studies that validated its prognostic use^(15)^. hs-CRP was obtained using the Multigent Vario Architect^®^ assay(Abbott Laboratories, Abbott Park, IL,USA), which is a latex immunoassay. The lower detection limit is 0.01mg/dL. The total variation coefficient in the assay is≤6%^(16)^. In turn, the TroponinI protein was quantified through an immunoassay. TroponinI is considered the gold standard among the biochemical markers for myocardial necrosis, presenting excellent sensitivity and specificity^(17)^. It is used for acute myocardial infarction diagnoses as well, as this biochemical test presents high prognostic value in these cases and is also useful for risk stratification in acute coronary syndromes. In addition, TroponinI can be employed to detect acute effects of myocardial lesions related to chemotherapy-induced toxicity^(18)^. A consensus in the Specialists Committee for the Food and Drug Administration concluded that TroponinI(cTnI) and TroponinT(cTnT) are sensitive, specific and robust heart failure biomarkers, allowing detecting and quantifying cell lesions and death related to testing new drugs^(18)^. The reference values for cardiac TroponinI are as follows: below 0.01mg/L(through the immunoenzymatic assay). In the case of TroponinI, the lower detection limit of the immunoassay with electrochemiluminescence used in the current study(<0.01mg/L) was used as a normality reference, according to the recommendation set forth in international studies targeted at chemotherapy-induced subclinical cardiotoxicity^(18)^.

The neutrophil-lymphocyte ratio (NLR) was calculated by dividing the absolute neutrophil and lymphocyte counts and was classified as high when ≥3.0 and as low when<3.0, as evidenced in studies conducted with patients with breast cancer and other types of solid tumors^(19-20)^. The platelet-lymphocyte ratio(PLR) was defined as high when >200, indicating persistent systemic inflammation and worse anti-tumor activity, as already shown in previous research studies^(21-23)^.

### Data sources

As a first step, the *Afecc*-HSRC clinical oncologists’ appointment schedules were analyzed to identify any potential patient with first-time indication for chemotherapy treatments. After their consultation with the clinical oncologist, each patient was referred to the Chemotherapy sector for scheduling purposes, when they were first approached to invite them to participate in the research, according to the eligibility criteria. The research objectives were presented in this initial approach and those who accepted signed a Free and Informed Consent From. Data collection was conducted between November2022 and December2023 and was in charge of Oncology nurses and undergraduate Nursing and Obstetrics students from *Universidade Federal do Espírito Santo*(UFES) – members of the Oncology Research and Study Group (*Grupo de Estudo e Pesquisa em Oncologia*,GEPONC)/National Council for Scientific and Technological Development(CNPq) – who were trained and supervised by the lead researchers. The training participants were Nursing students attending the “Semiology and Semiotechnics” and “Oncology Nursing” academic disciplines (taught by the lead researcher), as well as Oncology nurses, in order to organize and identify all data collectors. The training consisting in applying the project questionnaires and instruments in a group, as well as in simulations of the physical examinations and of standardized and proper use of electrocardiograms. A nutritionist that is an MSc in Nutrition and a GEPONC/CNPq member was invited to lead the simulations corresponding to the anthropometric measures.

The lead researchers prepared a sociodemographic and clinical questionnaire based on a systematic literature review on the topic and also on the clinical-epidemiological variables included in the Tumor Recording Form from the *Afecc*-HSRC Cancer Hospital Files. The medical records were consulted to obtain more detailed information about clinical issues and cancer treatments, as well as to access hemogram data. In addition to that, Nursing consultations were conducted, including collection of clinical histories, physical examination, anthropometric evaluation and electrocardiogram(ECG).

The following variables were assessed in the ECG analysis: QRS complex and corrected QT (QTc) interval duration^(24)^. According to the guidelines set forth by the Brazilian Society of Cardiology (*Sociedade Brasileira de Cardiologia*, SBC), a widened QRS is considered as lasting more than 120milliseconds (0.12seconds). The QT interval corresponds to the time elapsed between QRS complex initiation and T-wave end, representing the overall duration of the electrical ventricular activity. However, as the QT interval is directly influenced by cardiac frequency, a common practice is to use corrected QT(QTc) intervals, as calculated following Bazett’s formula. The reference values for QTc vary according to gender, with up to 450milliseconds(ms) and up to 470ms considered normal for men and women, respectively^(24)^.

The questionnaire data included the following: age, gender, self-declared skin color, schooling level, marital status, time since diagnosis at treatment initiation(in days), previous history and presence of comorbidities, tobacco and alcohol consumption, International Classification of Diseases (ICD-10) and staging. The physical examination included a cardiovascular examination (cardiac frequency, rhythm, amplitude, peripheral perfusion, jugular turgidity, limb edema, *ictuscordis* and cardiac auscultation) and an anthropometric evaluation(weight, height, abdominal circumference and waist circumference).

The cardiovascular examination consisted in inspection, palpation, percussion and auscultation, as standardized. The jugular vein was inspected to assess central venous pressure and identify jugular turgidity signals. In order to assess cardiac frequency(CF), the tips of the point and middle fingers were applied to the wrist anterolateral region and the radial pulse beats per minute were counted. The CF reference value adopted followed the Brazilian guideline, considering intervals between 50 and 99bpm as normal^(24)^. Palpation was also used to assess heart rhythm regularity and irregularity, as well as its amplitude, observing any abnormality. The *ictus cordis* was inspected and palpated in the thorax, between the fourth and fifth left intercostal spaces, at the left mid-clavicular line.

The cardiac auscultation procedure was performed with the aid of a double stethoscope (Bic^®^Eternity) that allowed identifying normal cardiac sounds (S1orS2) or even detecting abnormal ones such as whistling, ruptures or murmurs. A 12-lead electrocardiograph (Cardiocare 2000 12-channel Bionet^®^) was used for the ECGs. The participants lied down on a stretcher and took off their upper garments; subsequently, the collector placed the electrodes on their thorax (in the V1 to V6positions), wrists and ankles.

The anthropometric assessment data were collected in triplicate and the mean value was calculated through standardized measures. A scale (Omron^®^) with 150kg capacity and 0.1kg precision was used to weigh the participants, who stayed in a standing position on the device, bare-footed and with the fewest garments possible. As for height, the patients remained in a standing position, with their arms stretched along their body, and staring at the horizon; the measurement was taken using a portable stadiometer (Sanny^®^) with 1mm precision. An inextensible and inelastic tape measure (Cescorf^®^) was used to measure abdominal circumference(AC) and waist circumference(WC). WC was measured from the midpoint between the iliac crest and the last rib lower edge with the individuals in a standing position, their feet together, upper garments raised and arms crossed at the chest. Likewise, AC was measured at the umbilical scar height.

It is noted that all the biomarkers and ECGs were collected at the same moment for all participants; in other words, before the outpatient chemotherapy first session infusion, so as to minimize measuring bias.

### Sample size

For sample size calculation, the casuistic of the service where the patients were recruited was considered, in addition to by consulting previous studies conducted with patients in the aforementioned hospital^(25-27)^. Therefore, considering the estimated incidence of cardiotoxicity induced by chemotherapy agents at around 40%(p=0.40) based on the scientific literature^(28-29)^, sample size was calculated by defining α at 5%(TypeI error) and taking into account 80% statistical test power(β=0.20). In addition, 20% was added to the sample size in the calculation, considering probable losses/withdrawals. The formula used for sample calculation was the one initially proposed by Kish(1965): n=N.Z^2^.p.(1-p) / Z^2^.p.(1-p) + e^2^.N-1 (where n: Calculated sample, N: Population, Z: Normal variable, p: Actual probability of the event, and e: Sampling error)^(30)^.

Considering the population of diagnosed patients (only new ones) at *Afecc-Hospital Santa Rita de Cássia* in 2022 (n=3,513) without the novel coronavirus pandemic bias and defining α at 5%(sampling error), with a 95% confidence level and a minimum percentage of 80%(considering 20% losses), the samplen for this research was obtained:84patients.

### Data analysis

The categorical variables were presented by means of absolute and relative frequencies; in turn, the numerical ones were summarized using central tendency (mean and median) and dispersion (standard deviation and interquartile range) measures. Fisher’s Exact test was applied in the inferential analysis with the objective of verifying possible associations between the patients’ electrocardiographic parameters, inflammatory biomarkers and clinical and sociodemographic characteristics. According to the previously estimated minimum sample, the total number of participants considered was 84 during data analysis. However, some specific inferential analyses presented small sample variations(n<84) due to absence of punctual information in certain laboratory or electrocardiographic tests. Such missings were mainly due to technical flaws in processing samples, inconsistencies in records or collection unavailability on the same day of the clinical screening. A 5%(p<0.05) statistical significance level was considered. All the analyses were performed using the Rsoftware(version4.3.2) in the RStudio environment(version2023.09.1Build494).

### Ethical aspects

The study was approved by a Research Ethics Committee under CAAE56492222.6.0000.5060 and Opinion No.5,310,994. Due permission was also obtained from the hospital institution in the instances involved for data collection. After voluntarily stating their wish to take part in the study, the participants signed a Free and Informed Consent Form. It is noted that anonymity regarding the patients’ information was safeguarded by coding the data in the database, by removing personal identifiers and by restricting database access to only three researchers from the research team, in order to prioritize the confidentiality and privacy principles related to this research.

## Results

The subjects included were 84 patients with cancer diagnoses and a mean age of 57.6years old (SD=11.8). Most of the participants were women (n=61;72.62%), brown-skinned (n=34;40.48%) and married (n=43;51.19%). As for schooling level, 38.10%(n=32) and 30.95% (n=26) had Complete Elementary School and Complete High School,respectively.

The “Malignant neoplasm of breast” diagnosis accounted for 57.14%(n=48), followed by “Neoplasm of colon” with 22.62%(n=19) and by “Malignant neoplasm of bronchus and lung” with 5.95%(n=5). In relation to the tumors’ clinical staging, the most prevalent one was TypeII in 57.14%(n=48) followed by TypeIII(n=24;28.57%) and TypeI(n=9;10.71%). The patients’ sociodemographic and clinical characteristics are presented in Table 1.

**Table 1-:** Sociodemographic and clinical characterization corresponding to the cancer patients with outpatient chemotherapy indicated(n = 84). Vitória, ES, Brazil,2022-2023

**Variables**	**n**	**%**
**Age (in years old)**		
Mean (Standard Deviation)	57.68 (11.81)	-
Median (Interquartile Range)	58.00 (49.75 - 67.00)	-
**Age group**		
<50years old	21	25.00
50-64years old	36	42.86
≥65years old	27	32.14
**Gender**		
Male	23	27.38
Female	61	72.62
**Self-reported skin color/race**		
White	32	38.10
Black	10	11.90
Brown	34	40.48
Asian	3	3.57
No information	5	5.95
**Schooling**		
Illiterate	5	5.95
Incomplete Elementary School	9	10.71
Complete Elementary School	32	38.10
Incomplete High School	26	30.95
Complete High School	3	3.57
Incomplete Higher Education	1	1.19
Complete Higher Education	4	4.76
Graduate Studies	4	4.76
**Marital status**		
Single	15	17.86
Married	43	51.19
Widowed	11	13.10
Divorced	11	13.10
Stable union	4	4.76
ICD-10*		
C18 - Malignant neoplasm of colon	19	22.62
C50 - Malignant neoplasm of breast	48	57.14
C54.1 - Malignant neoplasm; Endometrium	1	1.19
C67 - Malignant neoplasm of bladder	2	2.38
C60 - Malignant neoplasm of penis	1	1.19
C34 - Malignant neoplasm of bronchus and lung	5	5.95
C31.9 - Malignant neoplasm of face sinuses	1	1.19
C160 - Malignant neoplasm: Cardia	1	1.19
C44 - Undifferentiated malignant neoplasm	1	1.19
C22.1 - Cholangiocarcinoma	1	1.19
C56 - Malignant neoplasm of ovary	1	1.19
C16 - Malignant neoplasm of stomach	1	1.19
C48 - Malignant neoplasm of retroperitoneum and peritoneum soft tissues	1	1.19
C15 - Malignant neoplasm of esophagus	1	1.19
**Treatment**		
Chemotherapy	48	57.14
Surgery + Chemotherapy	26	30.95
Chemotherapy + Hormone therapy and Radiotherapy	1	1.19
Chemotherapy + Radiotherapy	3	3.57
Chemotherapy + Surgery	6	7.14
**Staging**		
I	9	10.71
II	48	57.14
III	24	28.57
No information	3	3.57

*ICD-10 = International Classification of Diseases and Health-Related Problems-10

Table 2 presents all the information obtained from the cancer patients’ hemograms and biomarkers. As for the red series, the erythrocyte, hemoglobin and hematocrit mean values were 4.3millions/mm^3^, 12.53g/dl and 37.47%, respectively. In turn, the leukocyte and platelet mean counts were 7,380/mm^3^ and 312,000/mm^3^. The neutrophil-lymphocyte ratio(NLR) was 2,910/mm^3^, with most of the participants (n=62;76.54%) in the low classification. The mean platelet-lymphocyte ratio(PLR) was 175,850/mm^3^(SD=90.86) with most participants (n=59;72,84%) presenting low PLR values. In relation to the biomarkers, the mean for TroponinI was 3.23pg/mL and was only high in 1participant; in turn, the mean for C-reactive protein(CRP) was 6.83 mg/dL, with 30participants presenting high cardiovascular risk.

**Table 2-:** Hemogram, NLR*, PLR^†^, CRP^‡^ and Troponin values corresponding to the cancer patients with outpatient chemotherapy indicated(n = 84). Vitória, ES, Brazil,2022-2023

**Variables**	**n**	**%**
**Erythrocytes(millions/mm** ^3^ **)**		
Mean (Standard Deviation)	4.30 (0.56)	-
Median (Interquartile Range)	4.27 (3.94 - 4.60)	-
**Hemoglobin (g/dL)**		
Mean (Standard Deviation)	12.53 (1.69)	-
Median (Interquartile Range)	12.60 (11.60 - 13.50)	-
**Hematocrit (%)**		
Mean (Standard Deviation)	37.47 (4.67)	-
Median (Interquartile Range)	37.40 (35.30 - 40.50)	-
**Leukocytes (millions/mm** ^3^ **)**		
Mean (Standard Deviation)	7.38 (2.57)	-
Median (Interquartile Range)	6.95 (5.78 - 8.25)	-
**Platelets (millions/mm** ^3^ **)**		
Mean (Standard Deviation)	312.00 (108.47)	-
Median (Interquartile Range)	294.00 (237.00 - 365.00)	-
NLR* **(millions/mm** ^3^ **)**		
Mean (Standard Deviation)	2.91 (3.71)	-
Median (Interquartile Range)	1.96 (1.57 - 2.66)	-
NLR* **(millions/mm** ^3^ **)**		
Low	62	76.54
High	19	23.46
**PLR** ^†^ **(millions/mm** ^3^ **)**		
Mean (Standard Deviation)	175.81 (90.86)	-
Median (Interquartile Range)	147.92 (115.90 - 202.31)	-
**PLR** ^†^ **(millions/mm** ^3^ **)**		
Low	59	72.84
High	22	27.16
**TroponinI (pg/mL)**		
Mean (Standard Deviation)	3.23 (5.44)	-
Median (Interquartile Range)	1.50 (0.80 - 2.83)	-
**TroponinI (pg/mL)**		
Normal	83	98.81
High	01	1.19
**CRP** ^‡^ **(mg/dL)**		
Mean (Standard Deviation)	6.83 (18.84)	-
Median (Interquartile Range)	1.67 (0.68 - 4.41)	-
**CRP** ^‡^ **(mg/dL) (cardiovascular risk)**		
Low risk	30	35.71
Moderate risk	24	28.57
High risk	30	35.71

*NLR = Neutrophil-Lymphocyte Ratio; ^†^PLR = Platelet-Lymphocyte Ratio; ^‡^CRP = C-Reactive Protein

In relation to the chemotherapy agent classes, Table 3 allows observing that the Taxane agent + Alkylating agent + Anthracycline + Monoclonal antibody combination was the most frequent both among the patients without QTc prolongation(n=34) and among those with prolonged QTc(n=4). The Anti-metabolite + Platinum-derived combination was used in 2patients without QTc prolongation and in 1 with prolonged QTc; in turn, the Platinum-derived + Taxane agent combination was employed in 11patients without QTc prolongation and in 1 with prolonged QTc. The other combinations presented lower frequencies. There was no statistically significant association between the different combinations of chemotherapy agents and incidence of QTc prolongation(p=0.285).

As for the CRP levels, it was verified that most of the patients with prolonged QTc presented high CRP(n=6); in turn, the distribution across the categories was more balanced among those without QTc prolongation, namely: low(n=25), moderate(n=20) and high(n=23). Even so, no statistically significant association was identified between cardiovascular risk as estimated by CRP and presence of prolonged QTc(p=0.329).

In a general way, the analysis regarding the ECG association with CRP and the sociodemographic/clinical variables in Table 4 revealed absence of statistical significance for most of the variables under study. However, a significant association was in fact observed between waist circumference and CF(p=0.037), with tachycardia being more frequent among patients with increased risk for abdominal adiposity. Inverted T-wave and BMI were also significantly associated(p=0.005) and this finding was more common in individuals with BMI values above 20.5, which may suggest a relationship between overweight/obesity and alterations in ventricular repolarization.

**Table 3-:** Association of prolonged QTc* interval with chemotherapy agent classes and CRP ^†^ (cardiovascular risk)(n = 78). Vitória, ES, Brazil,2022-2023

**Variables**	Prolonged QTc* **interval**		**p** ^‡^
	**No**	Yes	
**Chemotherapy agent class**			0.285
Anti-metabolite + Platinum-derived	2	1	
Platinum-derived + Taxane agent	11	1	
Taxane agent + Alkylating agent + Anthracycline + Monoclonalantibody	34	4	
Taxane agent + Alkylating agent + Platinum-derived	1	1	
Anti-metabolite + Topoisomerase inhibitor + Platinum-derived + Taxaneagent + Folic acid analogue	20	3	
**CRP** ^†^ **(cardiovascular risk)**			0.329
Low	25	2	
Moderate	20	2	
High	23	6	

*QTc = Corrected QT; ^†^ CRP = C-Reactive Protein; ^‡^ p-value calculated based on Fisher’s Exact test

Table 5 presents the association of the participants’ previous history with ECG and CRP. There was a statistically significant association between tobacco consumption and heart rhythm(p=0.010), with higher frequency of irregular rhythm among smokers. The other electrocardiographic parameters (CF, QTc interval, ST elevation, inverted T-wave and widened QRS) did not present significant associations with the comorbidities evaluated(p>0.05). In relation to CRP, significant associations were observed with DM(p=0.033) and alcohol consumption(p=0.033), with high levels being more frequent among diabetics and drinkers.

**Table 4-:** Association of the ECG* parameters corresponding to cancer patients with outpatient chemotherapy indicated with CRP^†^ (cardiovascular risk) and sociodemographic and clinical variables(n = 78). Vitória, ES, Brazil,2022-2023

**Variables**	**Rhythm**				**CF** ^‡^					**Prolonged QTc** ^§^ **interval**				**Inverted T-wave**					**Widened QRS** ^||^		
	**Regular**	**Irregular**	p**		**Bradycardia**	**Normocardia (60-100)**	**Tachycardia**	p**		**No**	**Yes**	p**		**No**	**Yes**	**V1**	**p-value**		**No**	**Yes**	p**
**CRP** ^†^ **(cardiovascular risk)**			0.837					0.600				0.329					0.454				0.378
Low	26	1			4	23	0			25	2			24	3	0			25	2	
Moderate	20	1			3	18	1			20	2			20	1	1			21	1	
High	28	1			3	23	3			23	6			28	1	0			29	0	
**Gender**			0.066					0.302				0.134					0.727				0.190
Male	19	2			4	16	2			17	5			20	2	0			20	2	
Female	55	1			6	48	2			51	5			52	3	1			55	1	
**Age group**			0.340					0.701				0.434					0.280				0.471
<50years old	19	1			1	18	1			18	2			20	0	0			20	0	
50-64years old	32	0			4	27	2			30	3			31	2	0			32	1	
≥65years old	23	2			5	19	1			20	5			21	3	0			23	2	
**Self-reported skin color/race**			1.000					0.884				0.697					0.395				1.000
White	29	1			5	24	1			27	3			28	1	1			29	1	
Black	9	0			2	7	0			7	2			8	1	0			9	0	
Brown	28	2			3	25	3			27	4			29	2	0			29	2	
Asian	3	0			0	3	0			3	0			2	1	0			3	0	
No information	5	0			0	5	0			4	1			5	0	0			5	0	
**BMI** ^¶^			0.101					1.000				0.241					**0.005**				0.076
Below 20.5	1	1			0	2	0			1	1			0	2	0			1	1	
Above 20.5	73	2			10	62	4			67	9			72	3	1			74	2	
**Waist Circumference**			0.290					**0.037**				0.590					0.712				0.763
Normal	13	2			0	15	0			14	1			13	2	0			14	1	
Increased risk	20	0			6	14	0			16	4			19	1	0			20	0	
Very increased risk	41	1			4	35	4			38	5			40	2	1			41	2	
**Abdominal Circumference**			0.387					0.062				1.000					0.796				0.571
Normal	30	2			7	26	0			29	4			30	3	0			31	2	
Obese	44	1			3	38	4			39	6			42	2	1			44	1	
**Abdominal Circumference**			1.000					0.755				0.699					0.151				0.145
Normal	18	1			2	17	0			16	3			16	3	0			17	2	
Obese	56	2			8	47	4			52	7			56	2	1			58	1	
**Blood glucose**			0.425					0.014				0.398					0.450				0.576
Normal	30	1			5	27	0			30	2			30	2	0			30	2	
Pre-diabetic	13	2			2	11	2			13	2			12	2	1			14	1	
Diabetic	24	0			0	22	2			19	5			23	1	0			24	0	
No information	7	0			3	4	0			6	1			7	0	0			7	0	
**Staging**			0.738					0.306				0.143					0.199				1.000
I	8	0			2	6	0			7	1			7	1	0			8	0	
II	42	1			6	36	2			37	7			43	1	0			42	2	
III	22	2			2	21	1			23	1			20	3	1			23	1	
No information	2	0			0	1	1			1	1			2	0	0			2	0	
**Chemotherapy agent class**			0.118					0.076				0.285					0.381				0.301
Anti-metabolite + Platinum-derived	2	0			2	1	0			2	1			3	0	0			3	0	
Platinum-derived + Taxane agent	12	0			3	8	1			11	1			10	2	0			11	1	
Taxane agent + Alkylating agent + Anthracycline + Monoclonalantibody	3	1			2	35	1			34	4			37	1	0			38	0	
Taxane agent + Alkylating agent + Platinum-derived	2	0			0	2	0			1	1			2	0	0			2	0	
Anti-metabolite + Topoisomerase inhibitor + Platinum-derived + Taxane agent + Folic acid analogue	21	2			3	18	2			20	3			20	2	1			21	2	

*ECG = Electrocardiogram; ^†^CRP = C-Reactive Protein; ^‡^CF = Cardiac Frequency;^§^QTc = Corrected QT; ^||^QRS = QRS complex; ^¶^BMI=BodyMass Index; **p = p-value calculated based on Fisher’s Exact test

**Table 5-:** Association of the previous history corresponding to cancer patients with outpatient chemotherapy indicated with ECG* and C-reactive protein(cardiovascular risk)(n=78). Vitória, ES, Brazil,2022-2023

**Variables**		**SAH** ^†^				**AMI** ^‡^				**Dyslipidemia**				**Diabetes *Mellitus***				**Stroke**				**Valve disease**				**Tobacco consumption**				**Alcohol consumption**				**Family history ofcardiopathies**		
		**No**	**Yes**	**p** ^§^		**No**	**Yes**	**p** ^§^		**No**	**Yes**	**p** ^§^		**No**	**Yes**	**p** ^§^		**No**	**Yes**	**p** ^§^		**No**	**Yes**	**p** ^§^		**No**	**Yes**	**p** ^§^		**No**	**Yes**	**p** ^§^		**No**	**Yes**	**p** ^§^
**Rhythm**				0.793				1.000				1.000				0.582				1.000				1.000				**0.010**				1.000				1.000
Regular	39	35			70	4			65	9			60	14			71	3			73	1			70	3			63	10			25	48	
Irregular	1	2			3	0			3	0			2	1			3	0			3	0			1	1			2	0			1	2	
	No information	1	0			1	0			1	0			1	0			1	0			1	0			0	1			1	0			0	1	
**CF** ^||^				0.343				1.000				1.000				0.328				1.000				1.000				0.690				0.628				0.188
Bradycardia	7	3			10	0			9	1			8	2			10	0			10	0			9	1			9	1			1	9	
Normocardia (60-100)	33	31			60	4			56	8			53	11			61	3			63	1			58	4			54	8			23	40	
	Tachycardia	1	3			4	0			4	0			2	2			4	0			4	0			4	0			3	1			2	2	
**Prolonged QTc** ^¶^ **interval**				0.504				0.429				0.324				0.093				0.341				1.000				0.477				0.337				0.151
No	37	31			65	3			61	7			57	11			66	2			67	1			63	4			59	8			25	42	
	Yes	4	6			9	1			8	2			6	4			9	1			10	0			8	1			7	2			1	9	
ST** elevation				0.474				1.000				1.000				1.000				1.000				1.000				1.000				1.000				1.000
No	41	36			73	4			68	9			62	15			74	3			76	1			70	5			65	10			26	50	
	Yes	0	1			1	0			1	0			1	0			1	0			1	0			1	0			1	0			0	1	
**Inverted T-wave**				0.114				1.000				0.533				0.658				0.216				1.000				1.000				1.000				0.214
No	40	32			68	4			64	8			57	15			70	2			71	1			66	5			61	10			26	45	
Yes	1	4			5	0			4	1			5	0			4	1			5	0			4	0			4	0			0	5	
	V1	0	1			1	0			1	0			1	0			1	0			1	0			1	0			1	0			0	1	
**Widened QRS** ^††^				0.601				0.148				1.000				1.000				0.112				1.000				1.000				1.000				0.547
No	40	35			72	3			66	9			60	15			73	2			74	1			69	5			64	10			26	48	
Yes	1	2			2	1			3	0			3	0			2	1			3	0			2	0			2	0			0	3	
**C-reactive protein (cardiovascular risk)**				0.247				0.674				0.741				**0.033**				0.774				1.000				0.431				**0.033**				0.688
Low	13	14			26	1			25	2			25	2			26	1			27	0			24	1			25	0			9	18	
Moderate	15	7			20	2			19	3			19	3			22	0			22	0			19	3			18	4			6	16	
	High	13	16			28	1			25	4			19	10			27	2			28	1			28	1			23	6			11	17	

*ECG = Electrocardiogram; ^†^SAH = Systemic Arterial Hypertension; ^‡^AMI = Acute Myocardial Infarction; ^§^p = p-value calculated based on Fisher’s Exact test; ^||^CF = Cardiac Frequency; ^¶^QTc = Corrected QT; **ST = ST segment; ^††^QRS = QRS complex

## Discussion

Most of the participants included in the current study were women, with higher incidence of breast cancer diagnoses, followed by colon and bronchus and lung cancer. In this sense, the evidence corroborates that breast cancer is the tumor that most affects women worldwide, when excluding non-melanoma skin cancer^(31)^. Nevertheless, this distribution differs from the international^(32)^ and national^(33)^ estimates, which can be associated with socioeconomic/environmental factors and with other issues related to the profile of the population under study. Given this, a study shows that the number of malignant breast neoplasm cases has been progressively increasing as industrialization and urbanization advanced^(34)^.

The mean age (57.68 years old) is in line with the age profile commonly described for cancer patients^(33-34)^. By itself, aging is a risk factor for carcinogenesis, associated with comorbidities and with genomic instability such as telomerase mutations. Therefore, biological age is related to increased risk for any type of cancer^(35)^.

As for the therapies, prevalence of the Chemotherapy scheme consisting in taxanes, alkylating agents, anthracyclines and monoclonal antibodies was verified, usually recommended for the treatment of solid tumors such as in breast, bladder and lung cancer. These chemotherapy classes are recognized as one of the main agents with high cardiotoxic potential^(7,36)^.

A study conducted with early-stage breast cancer patients shows that the cardiotoxic effects exerted by Doxorubicine (DOX) can appear late in time, even years after chemotherapy exposure, highlighting the importance of early identifying at-risk individuals^(7,37)^. In addition to anthracyclines, Docetaxel and Cyclophosphamide also exert toxic effects on the myocardium, although their mechanisms are not yet fully elucidated. They are both associated with the risk of left ventricular dysfunction(LVD)^(38-40)^, which represents the main cardiotoxic manifestation^(41)^.

In the research, 23.46%(n=19) and 27.16%(n=22) of the patients presented high NLR and PLR baseline values, respectively. On the contrary, a study that included patients with breast cancer diagnoses undergoing treatment with Anthracycline did not identify any alteration in the NLR baseline values; however, an increase in this biomarker was in fact observed after chemotherapy exposure, associated with a four-fold risk of developing cardiac dysfunction, suggesting that Anthracycline exerts greater influence than the one caused by the disease itself^(40)^.

Another study (which related NLR and PLR to nutritional status, clinical/sociodemographic factors and quality of life among women hospitalized with non-metastatic breast cancer) verified high risk for people with average and high NLR and PLR values, which means low anti-tumor activity and worse prognoses^(14)^. Related to systemic inflammation, these biomarkers have been studied as accessible risk stratification tools^(41)^.

Also considered as an important inflammatory biomarker, CRP was high in 35.71%(n=30) of the patients, indicating increased cardiovascular risk. In addition, a systematic review showed that, along with Troponin and TypeB natriuretic peptides(BNPs), CRP is related to severe cardiovascular adverse outcomes such as myocardial infarction and heart failure^(42)^. An association between DM and CRP was observed in this study, indicating that inflammatory processes are more frequent in the patients. This condition contributes to increased cardiovascular risk, as well as to progression of vascular complications^(43)^.

Monitoring cardiotoxicity and systemic inflammation through dosage of serum biomarkers such as NLR, PLR, TroponinI and CRP is an accessible, minimally invasive and low-cost method that contributes to risk stratification and to early detecting this condition before the initial therapy, in addition to favoring early diagnosis of CVDs both during and after treatments, providing aids to detect cancer patients that can benefit from cardioprotective treatments or need long-term follow-up^(44-46)^.

Multiple pre-existing cardiovascular risk factors (such as hypertension, Diabetes *Mellitus*, dyslipidemias and obesity) are widely acknowledged in the literature as conditions that increase the risk of cardiovascular toxicity associated with cancer treatments^(36)^. However, there were no statistically significant associations between clinical/laboratory variables and most of the electrocardiographic parameters evaluated in the current sample. Despite that, the fact of having identified a relationship between BMI and inverted T-wave (p=0.005) stands out. Such finding is consistent with other studies reporting the impact exerted by high BMI values on the myocardium electrical function, favoring changes in repolarization^(47-48)^.

Even if QTc prolongation was not significantly associated with the variables researched, it was noticed that 10patients already presented this alteration at the baseline moment, reinforcing the need for continuous monitoring. Diverse evidence shows that QTc prolongation can be intensified throughout a given treatment, especially with cumulative use of anthracyclines and taxanes, thus increasing the risk of severe arrhythmias^(4,49)^.

Atrial fibrillation, ectopic ventricular beats and QTc prolongation itself are among the most frequent arrhythmias in cancer patients, with the possibility of worsening during treatment^(50-52)^. In this context, electrocardiographic evaluations should be systematically incorporated from the initial treatment phase.

It is important to consider that the absence of associations found in this study can be related to the fact that the patients were in the Pre-chemotherapy phase, a period during which the cardiotoxic effects associated with the treatment have not yet manifested themselves. Thus, although there are no significant associations at the baseline moment, it is important to note that these factors are well established in the literature as predictors of a higher risk of developing cardiotoxicity, especially in patients exposed to agents such as Anthracycline^(7,36)^.

In fact, previous studies show that individuals with these comorbidities were up to 12times more likely to develop anthracycline-induced cardiac dysfunction^(53)^. In addition to that, according to the Clinical Practice Guidelines proposed by the American Society of Clinical Oncology, the presence of multiple cardiovascular risk factors associated with using anthracyclines and age above 60years old represents a scenario characterized by a high risk of developing cardiac dysfunction^(54-55)^. Consequently, this analysis further demonstrates the impact exerted by comorbidities on cardiotoxic risk and points to the importance of controlling the underlying diseases, in addition to implementing evaluations and follow-up of patients in charge of a multi-disciplinary team targeted at the cardiovascular system from chemotherapy initiation to after such treatment as a prevention strategy, following the recommendations proposed by the Brazilian Cardio-Oncology Guideline^(36)^.

It is also important to explore the potential of ECGs and serum biomarkers such as NLR, PLR TroponinI and CRP as diagnostic and monitoring tools not only for cardiac alterations but also for cardiovascular risk stratification, aiming at early identifying cardiotoxicity and at selecting patients that may benefit from cardioprotective interventions^(41)^.

Although the inflammatory and cardiac biomarkers analyzed in this study possess acknowledged clinical relevance, it is important to consider that the assessment was performed in the Pre-chemotherapy phase, a period prior to the full manifestation of the most intense cytotoxic and inflammatory effects resulting from anti-neoplastic treatments. This can justify the absence of statistically significant associations between these markers and the clinical or electrocardiographic parameters evaluated. Although TroponinI’s prognostic usefulness is well established in subsequent treatment phases (especially as an anthracycline-induced ventricular dysfunction predictor)^(8,28)^, its low rise (only observed in one patient) limits its applicability as a baseline marker. Likewise, when used in isolation, high CRP (identified in 35.7% of the sample) presents low specificity as a cardiovascular risk marker, with the possibility of being influenced by other inflammatory, infectious or metabolic conditions such as nutritional status, presence of Diabetes *Mellitus* or tumor load^(15,42)^. Nevertheless, early detection of these biomarkers even at sub-clinical levels can contribute to individualized risk stratification and to guiding preventive strategies in Cardio-Oncology, especially among patients with modifiable comorbidities or risk factors. Such approach is supported by international guidelines and by the most recent evidence on biomarkers in monitoring cardiotoxicity^(41,54)^.

Health professionals (especially nurses) play a fundamental role in the clinical surveillance of cancer patients; therefore, it is indispensable that they stay alert at the monitoring and evaluation of serum biomarkers and of ECGs to early identify cardiovascular risks and stratify them, both during treatment and during follow-up). Given the well-known cardiotoxicity of various chemotherapy agents, timely interventions can minimize complications and improve prognoses^(7,36)^.

In this context, the increasing incorporation of Omic sciences into the Nursing clinical practice stands out^(56-58)^, reinforcing the importance of Precision Nursing^(51-52)^. Applying customized strategies based on specific biomarkers and on each patient’s individual characteristics contributes to more effective and personalized care, centered on the person and targeted at evidence-based clinical decision-making^(58-60)^.

Therefore, it is indispensable to adopt monitoring protocols combined with therapeutic interventions grounded on evidence, with the objectives of mitigating cardiotoxicity, reducing cardiovascular morbidity and mortality and, consequently, improving cancer patients’ prognoses^(60)^. International guidelines such as those proposed by the European Society of Cardiology along with the European Society for Medical Oncology(ESMO), as well as the American Society of Clinical Oncology(ASCO), reinforce the need for structured and systematic follow-up in the case of patients exposed to cardiotoxic agents. Adopting these recommendations enables early detection of sub-clinical myocardial dysfunction, allowing timely and effective interventions to prevent evolution to heart failure and other adverse cardiovascular events^(7)^.

The findings of this study present relevant implications for the Nursing clinical practice, especially in the Oncology Nursing context. Identifying inflammatory and cardiac biomarkers already in the Pre-chemotherapy phase (even if at sub-clinical levels) evidences the need for Nursing teams’ proactive performance in cardiovascular risk early stratification^(60-61)^. For being accessible and low-cost, markers such as NLR, PLR, TroponinI and CRP can be incorporated as complementary tools to anamnesis and to physical examinations in cardiovascular screening, supporting the planning of personalized interventions and continuous surveillance throughout a given cancer treatment. Associated with a critical interpretation of biomarkers, systematically performing tests such as ECGs strengthens the role of Nursing personnel in early detecting cardiovascular alterations, in communication with multi-professional teams and in adopting cardioprotective strategies.

In addition, integrating these practices into the initial evaluation protocol for cancer patients favors comprehensive, safe and evidence-based care, in line with the Precision Nursing principles and with national and international Cardio-Oncology guidelines. Such evidence reinforces the importance of Nursing as playing a leading role in screening, monitoring and cardiovascular health education in Oncology care, directly contributing to preventing health problems and to improving these patients’ clinical outcomes.

The study limitations include the following: I. The discrete TroponinI rise (only detected in one patient), which limits interpretation regarding applicability of this marker in baseline conditions; II. The study was conducted in a single center specialized in cancer treatments and with a relatively small and heterogeneous sample, which may impair its external validity and generalization of its findings to other population groups or clinical contexts; III. Although the total number of patients reached the minimum estimated sample(n=84), some specific analyses presented punctual sample losses due to inconsistency or absence of laboratory and/or electrocardiographic data, which reduced the number of participants in certain variables and may have affected the statistical power of these analyses; IV. Absence of a Control Group comprised by cancer patients not subjected to chemotherapy or without cardiovascular risk factors; V. The study cross-sectional design, which precludes longitudinal follow-up of inflammatory and electrocardiographic alterations throughout a given cancer treatment; VI. Not having performed important complementary tests, such as NT-ProBNP dosage or echocardiograms, which might have contributed to more encompassing cardiac function evaluations; and VII. Having used CRP as an isolated marker in cardiovascular risk stratification, which limits its clinical applicability, especially in the absence of other complementary parameters.

However, our study does present relevant aspects despite its limitations, namely: I.Having assessed subjective and objective measures(through biomarker dosage); II.Standardized collection of clinical biomarkers and data (before the first chemotherapy session infusion), ensuring data reliability and validity; and III. Clinical relevance of the results for early cardiovascular risk stratification, aiming at well-being and better prognoses among cancer patients undergoing chemotherapy. However, we emphasize that the data presented offer important preliminary evidence about baseline cardiovascular evaluations in cancer patients at the Pre-chemotherapy phase, contributing to grounding future multi-center and longitudinal research studies.

It is suggested to conduct well-designed longitudinal studies with representative samples to understand the trends in cancer diagnoses over time and identify the associated cardiac risk factors during follow-up. In addition to that, prevention and intervention strategies targeted at mitigating the cardiac impairment risks resulting from chemotherapy treatments should be researched, with a focus on Cardio-Oncology and on Precision Nursing.

## Conclusion

In the current study, the absence of statistically significant associations across most of the parameters assessed can be related to the fact that the patients were at the Pre-chemotherapy phase, a period prior to onset of the expected cardiotoxic effects. Even so, it was possible to identify relevant cardiovascular risk factors such as systemic inflammation and high BMI, which along with certain chemotherapy protocols can potentiate the cardiac dysfunction risk.

These findings reinforce the need to incorporate systematic cardiovascular screening routines before the first chemotherapy infusion (in other words, from cancer follow-up initiation) as an essential strategy to detect early cardiovascular alterations and implement preventive, customized and timely interventions.

## Data Availability

All data generated or analysed during this study are included in this published article.
