# Supplementary material for: Chemotherapy-induced cardiotoxicity in cancer patients: an observational study at an Oncology reference hospital from southeastern Brazil
Source: Rev Lat Am Enfermagem. 2026 Jan 19;34:e4774. doi: 10.1590/1518-8345.7472.4774 (PMC12854660; doi:10.1590/1518-8345.7472.4774)
Supplement: Translation

# Translation: Cardiotoxicidade induzida por quimioterápicos em pacientes com câncer: estudo observacional em hospital de referência oncológica no Sudeste do Brasil*

**DOI:** 10.1590/1518-8345.7472.4775

**Authors:** Karoline Neumann, Karolini Zuqui Nunes, Wesley Rocha Grippa, Naira Santos D’Agostini, Sara Isabel Pimentel de Carvalho Schuab, Luís Carlos Lopes-Júnior

**Affiliations:** 1 Universidade Federal do Espírito Santo, Vitória, ES, Brasil. Universidade Federal do Espírito Santo ES Vitória Brasil; 2 Bolsista da Fundação de Amparo à Pesquisa e Inovação do Espírito Santo (FAPES), Brasil. Bolsista da Fundação de Amparo à Pesquisa e Inovação do Espírito Santo Brasil; 3 Universidade Federal do Espírito Santo, Departamento de Enfermagem, Vitória, ES, Brasil. Universidade Federal do Espírito Santo Departamento de Enfermagem ES Vitória Brasil; 4 Bolsista do Conselho Nacional de Desenvolvimento Científico e Tecnológico (CNPq), Brasil. Bolsista do Conselho Nacional de Desenvolvimento Científico e Tecnológico Brasil

**Keywords:** Neoplasias, Antineoplásicos, Fatores de Risco de Doenças Cardíacas, Biomarcadores, Eletrocardiograma, Cardiotoxicidade

## Abstract

avaliar o perfil clínico, inflamatório e eletrocardiográfico de pacientes oncológicos na fase pré-quimioterapia, visando à identificação precoce de sinais indicativos de toxicidade cardiovascular.

estudo observacional, transversal, realizado entre novembro de 2022 e dezembro de 2023, com adultos com câncer em estágios I a III, atendidos em um hospital do Sudeste brasileiro. Foram coletados dados sociodemográficos, clínicos e tumorais, além de exames laboratoriais e eletrocardiográficos realizados antes da primeira infusão quimioterápica. Análises descritivas foram conduzidas no *software* R.

participaram 84 pacientes, sendo a maioria mulheres (72,6%) com diagnóstico de câncer de mama (57,1%). Verificou-se prevalência de inflamação sistêmica, com proteína C-reativa elevada em 35,7%, razão neutrófilo-linfócito aumentada em 23,5% e razão plaqueta-linfócito em 27,2%. Observou-se associação significativa entre índice de massa corporal elevado e alterações na onda T (p=0,005). Embora 10 pacientes apresentassem intervalo QT corrigido prolongado, não foram identificadas outras associações estatisticamente significativas.

a fase pré-quimioterapia revelou alterações inflamatórias e eletrocardiográficas relevantes, mesmo na ausência de cardiotoxicidade estabelecida. Os achados reforçam a importância da avaliação cardiovascular basal e do acompanhamento multiprofissional na cardio-oncologia, especialmente em pacientes com fatores de risco modificáveis. Estudos longitudinais são recomendados para elucidar as trajetórias clínicas e prognósticas dessas alterações.

## Destaques

**(1)** Pacientes com antraciclinas e taxanos têm maior risco de cardiotoxicidade. **(2)** A proteína C-reativa esteve aumentada na primeira infusão de quimioterapia. **(3)** RNL e RPL elevados em até 27% dos pacientes antes do início do tratamento. **(4)** A estratificação precoce de risco possibilita o manejo da cardiotoxicidade. **(5)** Uso de biomarcadores amplia o potencial da Enfermagem de Precisão no cuidado personalizado.

## Introdução

Globalmente, as doenças e agravos não transmissíveis (DANT) configuram-se como as principais responsáveis pela morbidade e mortalidade da população. Este dado é impulsionado pela transição demográfica que contribui para o processo do envelhecimento da população, associada à transição epidemiológica marcada pela mudança nos padrões de doenças e mortalidade, sendo considerado um importante desafio em nível global^(1)^. Dentre as DANT o grupo das doenças crônicas não transmissíveis (DCNT) possuem destaque pela mortalidade no mundo, sendo as doenças cardiovasculares (DCV) as principais causas de morte, responsáveis por quase 18 milhões de óbitos^(2)^ seguido pelas neoplasias, correspondendo a quase 10 milhões de mortes. Na maioria dos países, o câncer é a primeira ou segunda principal causa de morte antes dos 70 anos de idade^(3)^.

Nos últimos anos, houve significativos avanços no tratamento do câncer com descobertas de novas drogas e terapias, que melhoram os prognósticos dos pacientes oncológicos^(4-5)^. No entanto, apesar dos benefícios, as doenças cardiovasculares emergiram como um dos principais efeitos adversos da terapêutica antineoplásica, contribuindo para a morbimortalidade dos pacientes^(4-6)^. Embora os quimioterápicos desempenhem um importante papel para os desfechos desses pacientes, o uso de algumas classes de quimioterápicos culminou em casos crescentes de efeitos secundários e de complicações cardiovasculares, sendo o desenvolvimento de cardiotoxicidade induzida por quimioterápico a ocorrência mais comum, que se estende após o tratamento^(6)^.

A Sociedade Europeia de Cardiologia (ESC) define a toxicidade cardiovascular (CV) como uma lesão cardíaca tanto funcional quanto estrutural que esteja condicionada ao tratamento oncológico (quimioterapia, radioterapia ou o próprio câncer)^(7-8)^. Ademais, a toxicidade CV pode ser dividida em aguda, subaguda ou tardia, em que os sinais e sintomas dessa toxicidade podem se apresentar em alguns anos após o tratamento do paciente com câncer, acometendo as estruturas cardíacas e gerando a insuficiência cardíaca (IC), doença arterial coronariana, doença cardíaca valvular, arritmias, doença de condução cardíaca e pericárdica^(8)^.

A cardiotoxicidade, no início, significava a queda da fração de ejeção, entretanto, a I Diretriz Brasileira de Cardio-Oncologia da Sociedade Brasileira de Cardiologia ressignificou tal condição como: cardiomiopatia com diminuição da fração de ejeção do ventrículo esquerdo (FEVE); sintomas da IC; sinais condicionados à IC; redução da FEVE de 5% a menos de 55% quando comparado ao valor basal e apresentando sinais ou sintomas da IC, ou diminuição de 10% da FEVE a menos de 55% sem sinais ou sintomas simultâneos^(9-10)^.

O surgimento da toxicidade cardíaca durante a terapia do câncer pode limitar o tratamento, levando à interrupção ou cessação do tratamento e potencialmente piorando os desfechos clínicos dos pacientes^(11)^. A monitorização de doenças cardíacas e de fatores de risco é fundamental e capaz de melhorar o prognóstico dos pacientes^(7)^.

Apesar dos avanços recentes na compreensão da cardiotoxicidade induzida por antineoplásicos, a maioria dos estudos concentra-se em desfechos clínicos tardios ou em populações já submetidas a múltiplos ciclos de tratamento, deixando uma lacuna importante sobre o perfil cardiovascular basal de pacientes oncológicos antes do início da quimioterapia. Conforme aponta uma revisão sistemática^(12)^, embora exista ampla documentação sobre a toxicidade cardíaca em pacientes com câncer hematológico, há escassez de estudos que investiguem alterações inflamatórias e eletrocardiográficas precoces em populações com neoplasias sólidas, especialmente em contextos da América Latina e em serviços públicos de referência oncológica.

Nesse sentido, nosso estudo se diferencia por avaliar, de forma original, marcadores inflamatórios e alterações eletrocardiográficas na fase pré-quimioterapia em pacientes com câncer em estágios iniciais a intermediários (I a III), contribuindo para a detecção precoce de possíveis sinais de cardiotoxicidade ainda antes da exposição acumulada à terapia antineoplásica. Trata-se, portanto, de um estudo original que busca ampliar a compreensão da fase basal da jornada cardio-oncológica, com potencial impacto na estratificação de risco e no planejamento de cuidados multiprofissionais. Nesse contexto, o objetivo do trabalho foi avaliar o perfil clínico, inflamatório e eletrocardiográfico de pacientes oncológicos na fase pré-quimioterapia, visando à identificação precoce de sinais indicativos de toxicidade cardiovascular.

## Método

### Desenho do estudo

Estudo observacional transversal, conduzido de acordo com as diretrizes do STROBE (*Strengthening the Reporting of Observational Studies in Epidemiology*)^(13)^ para garantir a transparência e a qualidade na apresentação dos dados e dos resultados.

### Cenário

Esta pesquisa foi realizada no Afecc-Hospital Santa Rita de Cássia (HSRC), único Centro de Alta Complexidade em Oncologia (CACON) do Espírito Santo, Brasil - referência no estado em tratamento oncológico para todo o Espírito Santo, sul da Bahia, leste de Minas Gerais e norte do Rio de Janeiro^(14)^ - no período de novembro de 2022 a dezembro de 2023.

### Participantes

Foram selecionados indivíduos de ambos os sexos com idade acima de 18 anos, que tinham diagnóstico anatomopatológico de neoplasia maligna em estágio I, II ou III da doença, independentemente do tipo de tumor, e apenas novos casos de pacientes submetidos a quimioterapia ambulatorial. Foram excluídos os participantes em estágio IV da doença, aqueles que recebiam cuidados paliativos exclusivos, assim como aqueles com mais de um tumor primário.

### Variáveis

Os dados sociodemográficos e a caracterização do tumor foram obtidos exclusivamente por meio de prontuários e da aplicação de questionário semiestruturado. Foi realizada uma coleta de sangue para avaliação de biomarcadores: hs-PCR e Troponina I (variáveis desfecho). As análises dos biomarcadores foram realizadas no Laboratório Tomasi, localizado no Afecc-HSRC. Salienta-se que o hemograma era rotina do hospital e, portanto, as informações foram extraídas diretamente do prontuário médico dos pacientes. Para a coleta de sangue, cada participante doou uma amostra de sangue venoso periférico (4 ml), coletado por meio de punção venosa na fossa cubital. Para isso, foram utilizados tubos a vácuo *Vacutainer*^®^*ethyldiamine tetra-acetic acid* (EDTA) [Becton Dickinson (BD), Franklin Lakes, NJ, EUA]. Para obtenção do plasma, as amostras biológicas coletadas foram processadas seguindo o seguinte protocolo: centrifugando-se os tubos *Vacutainer* contendo sangue por 10 minutos a 4°C, 581,2 g/2.000 r.p.m. (*Eppendorf Centrifuge* 5810R). Posteriormente, foi aliquotado com uma pipeta e o plasma e, então, transferido para microtubos de estocagem. Todo o material aliquotado foi armazenado no freezer a -80°C até a etapa de quantificação do biomarcador.

Os pontos de corte adotados para os biomarcadores foram definidos com base em diretrizes clínicas e literatura científica atualizada. Para a proteína C-reativa de alta sensibilidade (hs-PCR), utilizou-se a classificação da *American Heart Association* (AHA), que categoriza o risco cardiovascular como: baixo risco (<1 mg/dL), risco moderado (1–3 mg/dL) e alto risco (>3 mg/dL), com respaldo em estudos clínicos e laboratoriais que validaram seu uso prognóstico^(15)^. A hs-PCR foi obtida usando o ensaio *Multigent Vario Architect*^®️^ (Abbott Laboratories, Abbott Park, IL, EUA), que é um imunoensaio de látex. O limite inferior de detecção é de 0,01 mg/dL. O coeficiente de variação total do ensaio é ≤ 6%^(16)^. Já a quantificação da proteína troponina I foi por imunoensaio. A troponina I é considerada o padrão-ouro entre os marcadores bioquímicos de necrose miocárdica, apresentando excelente sensibilidade e especificidade^(17)^. É utilizada ainda, para o diagnóstico de infarto agudo do miocárdio, uma vez que este teste bioquímico tem alto valor prognóstico nesses casos e ainda serve para a estratificação de risco nas síndromes coronarianas agudas. Em adição, a troponina I serve para detectar efeitos agudos da lesão miocárdica relacionada à toxicidade devido à quimioterapia^(18)^. Um consenso do Comitê de Especialistas para o *Food and DrugAdministration*, concluiu que troponina I (cTnI) e troponina T (cTnT) são biomarcadores sensíveis, específicos e robustos de insulto cardíaco, permitindo a detecção e quantificação de lesões celulares e morte relacionadas ao teste de novas drogas^(18)^. Os valores de referência para troponina I cardíaca são respectivamente: inferior a 0,01 mg/L (pelo método de ensaio imunoenzimático). No caso da troponina I, adotou-se o limite inferior de detecção do imunoensaio com eletroquimioluminescência utilizado no presente estudo (<0,01 mg/L) como ponto de referência para normalidade, conforme recomendação de estudos internacionais voltados à cardiotoxicidade subclínica induzida por quimioterapia^(18)^.

A razão neutrófilo-linfócito (RNL) foi calculada pela divisão entre as contagens absolutas de neutrófilos e linfócitos, sendo classificada como elevada quando ≥3.0, e baixa quando <3.0, conforme evidenciado em estudos com pacientes com câncer de mama e outros tumores sólidos^(19-20)^. A razão plaqueta-linfócito (RPL) foi definida como elevada quando >200, indicando inflamação sistêmica persistente e pior atividade antitumoral, conforme demonstrado por pesquisas prévias^(21-23)^.

### Fontes de dados

Inicialmente, foram analisadas as agendas dos médicos oncologistas clínicos do Afecc-HSRC, para verificar a identificação de potenciais pacientes com indicação de primeira vez para tratamento quimioterápico. Após a consulta com o oncologista clínico, o paciente foi encaminhado para o setor de quimioterapia para agendamento e, neste momento, ocorreu a abordagem inicial do paciente para convidá-lo a participar da pesquisa, conforme os critérios de elegibilidade. Nesta abordagem inicial, foram apresentados os objetivos da pesquisa e, para os que aceitaram, procedeu-se à assinatura do Termo de Consentimento Livre e Esclarecido. A coleta de dados ocorreu entre novembro de 2022 e dezembro de 2023 e foi conduzida por enfermeiros oncologistas e estudantes de Graduação em Enfermagem e Obstetrícia da Universidade Federal do Espírito Santo (UFES) – membros do Grupo de Estudo e Pesquisa em Oncologia (GEPONC)/Conselho Nacional de Desenvolvimento Científico e Tecnológico (CNPq) – os quais foram capacitados e supervisionados pelos pesquisadores principais. Participaram do treinamento os estudantes de enfermagem que tivessem cursado as disciplinas de “Semiologia e Semiotécnica” e de “Enfermagem em Oncologia” – que foi ofertada pelo pesquisador principal – e também os enfermeiros oncologistas a fim de organizar e balizar todos os coletadores dos dados. O treinamento consistiu na aplicação em grupo dos questionários e instrumentos do projeto, bem como em simulações dos exames físicos e do uso padronizado e correto do eletrocardiograma. Foi convidada uma nutricionista, Mestra em Nutrição e membra do GEPONC/CNPq, para conduzir as simulações das medidas antropométricas.

Um questionário sociodemográfico e clínico foi elaborado pelos pesquisadores principais com base em revisão sistemática de literatura sobre o tópico, como também se baseando nas variáveis clínico-epidemiológicas contidas na Ficha de Registro Tumor do Registro Hospitalar de Câncer do Afecc - HSRC. Foram acessados os prontuários médicos para obter informações mais detalhadas sobre questões clínicas, do tratamento oncológico e para acessar os dados do hemograma. Além disso, a consulta de enfermagem foi realizada, com coleta do histórico clínico, exame físico, avaliação antropométrica e realização do eletrocardiograma (ECG).

Na análise do ECG, foram avaliadas as seguintes variáveis: duração do complexo QRS e intervalo QT corrigido (QTc)^(24)^. De acordo com as Diretrizes da Sociedade Brasileira de Cardiologia (SBC), considera-se um QRS alargado quando sua duração é superior a 120 milissegundos (0,12 segundos). O intervalo QT corresponde ao tempo entre o início do complexo QRS e o final da onda T, representando a duração total da atividade elétrica ventricular. No entanto, como o intervalo QT é diretamente influenciado pela frequência cardíaca, é comum utilizar o intervalo QT corrigido (QTc), calculado pela fórmula de Bazett. Os valores de referência para o QTc variam conforme o sexo, sendo considerados normais até 450 milissegundos (ms) para homens e até 470 ms para mulheres^(24)^.

Os dados do questionário incluíram: idade, sexo, cor autorreferida, escolaridade, estado civil, tempo do diagnóstico ao início do tratamento (em dias), história pregressa e presença de comorbidades, uso de tabaco, uso de bebida alcoólica, Classificação Internacional de Doenças (CID-10) e estadiamento. O exame físico incluiu o exame cardiovascular (frequência cardíaca; ritmo; amplitude; perfusão periférica; turgência jugular; edema de membros; *ictus cordis* e ausculta cardíaca) e avaliação antropométrica (peso, altura, circunferência abdominal e circunferência da cintura).

O exame cardiovascular deu-se pela inspeção, palpação, percussão e ausculta, conforme padronização. A inspeção da veia jugular foi realizada para avaliar a pressão venosa central e identificar sinais de turgência de jugular. Para avaliação da frequência cardíaca (FC) utilizou-se as polpas digitais dos dedos indicador e médio na região ântero-lateral do punho e mediu-se a quantidade de batimentos por minuto do pulso radial. O valor de referência da FC adotado seguiu a Diretriz Brasileira, sendo considerado normal o intervalo entre 50 e 99 bpm^(24)^. Através da palpação avaliou-se também a regularidade e irregularidade do ritmo cardíaco e amplitude, observando qualquer anormalidade. O *ictus cordis* foi inspecionado e palpado no tórax entre o quarto e o quinto espaço intercostal esquerdo, ao nível da linha médio-clavicular esquerda.

A ausculta cardíaca foi realizada com o auxílio de um estetoscópio duplo (Bic^®^*Eternity*) que permitiu a identificação de bulhas cardíacas normais (B1 ou B2) ou, ainda, a detecção de sons anormais como murmúrios, estalidos ou sopros. Para o ECG, foi utilizado um eletrocardiógrafo (Cardiocare 2000 12 Canais Bionet^®^) com 12 derivações, em que os participantes se deitavam na maca e removiam a parte de cima da roupa e o coletador posicionava os eletrodos no tórax (nas posições de V1 a V6), punhos e tornozelos.

A avaliação antropométrica foi coletada em triplicata e calculada a média, através de medidas padronizadas. Para o peso, utilizou-se a balança (Omron^®^) com capacidade para 150 Kg e precisão de 0,1 Kg, em que os participantes ficaram em posição ereta sobre o aparelho, descalços e com o mínimo de roupas possível. Para a altura, os pacientes permaneciam em posição ereta, braços estendidos ao longo do corpo e fixado o olhar no horizonte, e a medida foi realizada com o estadiômetro portátil (Sanny^®^) com precisão de 1 mm. Para medir a circunferência abdominal (CA) e circunferência da cintura (CC), usou-se uma fita métrica inextensível e inelástica (Cescorf^®^). A CC foi medida a partir do ponto médio entre a crista ilíaca e a borda inferior da última costela com os indivíduos eretos, pés juntos, roupa superior levantada e braços cruzados na região do peito. Do mesmo modo, a CA foi medida na altura da cicatriz umbilical.

Ressalta-se que todos os biomarcadores e o ECG foram coletados no mesmo momento para todos os pacientes, ou seja, antes da infusão da primeira sessão de quimioterapia ambulatorial, de modo a minimizar o viés de mensuração.

### Tamanho amostral

Para o cálculo do tamanho da amostra foi considerada a casuística do serviço onde os pacientes foram recrutados e a partir de estudos prévios conduzidos com pacientes no referido Hospital^(25-27)^. Para tanto, considerando-se a incidência estimada de cardiotoxicidade induzida por quimioterápicos com base na literatura científica ao redor de 40%^(28-29)^ (p=0,40), o número amostral foi calculado, fixando-se α em 5% (erro do tipo I) e levando-se em conta um poder de teste estatístico de 80% (β=0.20). Ademais, no cálculo foi adicionado 20% ao tamanho da amostra considerando a probabilidade de perdas/desistências. A fórmula utilizada para o cálculo amostral foi inicialmente proposta por Kish (1965): n=N.Z^2^.p.(1-p) / Z^2^.p.(1-p) + e^2^.N-1 (n: amostra calculada, N: população, Z: variável normal, p: real probabilidade do evento, e: erro amostral)^(30)^.

Considerando a população de pacientes diagnosticados (apenas casos novos) no Afecc-Hospital Santa Rita de Cássia em 2022 (n=3513) sem o viés da pandemia do novo coronavírus, e fixando α em 5% (erro amostral), com nível de confiança de 95%, e um percentual mínimo de 80% (considerando 20% de perdas), obteve-se o n amostral dessa pesquisa igual a 84 pacientes.

### Análise dos dados

As variáveis categóricas foram apresentadas por meio de frequências absolutas e relativas, enquanto as variáveis numéricas foram resumidas utilizando medidas de tendência central (média e mediana) e dispersão (desvio-padrão e intervalo interquartílico). Para a análise inferencial, foi aplicado o teste exato de Fisher com o objetivo de verificar possíveis associações entre os parâmetros eletrocardiográficos, biomarcadores inflamatórios e características clínicas e sociodemográficas dos pacientes. Durante a análise dos dados, o número total de participantes considerado foi de 84, conforme amostra mínima estimada previamente. Contudo, algumas análises inferenciais específicas apresentaram pequenas variações amostrais (n<84) devido à ausência pontual de informações em determinados exames laboratoriais ou eletrocardiográficos. Tais ausências decorreram, principalmente, de falhas técnicas no processamento de amostras, inconsistência nos registros ou indisponibilidade de coleta no mesmo dia da triagem clínica. Considerou-se o nível de significância estatística de 5% (p < 0,05). Todas as análises foram realizadas utilizando o *software* R (versão 4.3.2) no ambiente RStudio (versão 2023.09.1 Build 494).

### Aspectos éticos

O estudo foi aprovado pelo Comitê de Ética em Pesquisa, CAAE: 56492222.6.0000.5060, nº do Parecer 5.310.994. Assim, também, foi obtida a permissão da instituição hospitalar nas instâncias envolvidas para a coleta de dados. Os participantes, após manifestarem voluntariamente seu desejo de participar do estudo, assinaram o Termo de Consentimento Livre e Esclarecido. Ressalta-se que o anonimato das informações dos participantes foi protegido através da codificação dos dados no banco de dados, da remoção de identificadores pessoais e do acesso ao banco restritamente a um número de três pesquisadores que compunham a equipe de pesquisa, a fim de priorizar os princípios de confidencialidade e privacidade relacionados a esta investigação.

## Resultados

Foram incluídos um total de 84 pacientes diagnosticados com câncer, cuja média de idade era 57,6 anos e desvio-padrão de 11,8 anos. A maioria dos participantes eram mulheres (n=61; 72,62%), de cor parda (n=34; 40,48%) e casados (n=43; 51,19%). Quanto ao grau de instrução, 38,10% (n=32) possuíam o ensino fundamental completo e 30,95% (n=26) ensino médio incompleto.

O diagnóstico de neoplasia maligna da mama compreendeu 57,14% (n=48), acompanhado da neoplasia de cólon com 22,62% (n=19) e neoplasia maligna de brônquios e pulmões com 5,95% (n=5). Em relação ao estadiamento clínico do tumor, o mais prevalente foi o II presente em 57,14% (n=48) dos participantes, seguido do tipo III (n=24; 28,57%) e I (n=9; 10,71%). As características demográficas e clínicas dos pacientes estão apresentadas na Tabela 1.

**Tabela 1-:** Caracterização sociodemográfica e clínica dos pacientes oncológicos indicados à quimioterapia ambulatorial (n = 84). Vitória, ES, Brasil, 2022-2023

**Variável**		**n**	**%**
**Idade (em anos)**		
Média (Desvio-Padrão)	57,68 (11,81)	-
Mediana (Intervalo Interquartílico)	58,00 (49,75 - 67,00)	-
**Faixa etária**		
< 50 anos	21	25,00
50-64 anos	36	42,86
≥ 65 anos	27	32,14
**Sexo**		
Masculino	23	27,38
Feminino	61	72,62
**Cor autorreferida**		
Branca	32	38,10
Negro	10	11,90
Pardo	34	40,48
Amarelo	3	3,57
Sem Informação	5	5,95
**Escolaridade**		
Analfabeto	5	5,95
Fundamental incompleto	9	10,71
Fundamental completo	32	38,10
Médio incompleto	26	30,95
Médio completo	3	3,57
Superior incompleto	1	1,19
Superior completo	4	4,76
Pós-graduação	4	4,76
**Estado civil**		
Solteiro	15	17,86
Casado	43	51,19
Viúvo	11	13,10
Divorciado	11	13,10
União estável	4	4,76e
CID-10*		
C18 – Neoplasia maligna do cólon	19	22,62
C50 - Neoplasia maligna da mama	48	57,14
C54.1 – Neoplasia maligna de endométrio	1	1,19
C67 - Neoplasia maligna de bexiga	2	2,38
C60 - Neoplasia maligna em pênis	1	1,19
C34 - Neoplasia maligna de brônquios e pulmões	5	5,95
C31.9 Neoplasia maligna em seios da face	1	1,19
C160 - Neoplasia da cárdia	1	1,19
C44 - Neoplasia maligna indiferenciada	1	1,19
C22.1 - Colangiocarcinoma	1	1,19
C56 – Neoplasia maligna do ovário	1	1,19
C16 - Neoplasia maligna do estômago	1	1,19
C48 - Neoplasia maligna dos tecidos moles do retroperitônio e do peritônio	1	1,19
C15 - Neoplasia maligna do esôfago	1	1,19
**Tratamento**		
Quimioterapia	48	57,14
Cirurgia + quimioterapia	26	30,95
QT ^†^ + hormonoterapia e radioterapia	1	1,19
Quimioterapia + radioterapia	3	3,57
Quimioterapia + cirurgia	6	7,14
**Estadiamento**		
I	9	10,71
II	48	57,14
III	24	28,57
Sem Informação	3	3,57

*CID-10 = Classificação Internacional de Doenças e Problemas Relacionados à Saúde - 10; ^†^QT = Quimioterapia

A Tabela 2 apresenta as informações obtidas a partir do hemograma e biomarcadores dos pacientes oncológicos. No que se refere à série vermelha, a média das hemácias foi de 4,3 milhões/mm^3^, hemoglobina 12,53 g/dl e hematócrito 37,47%. Já a média dos leucócitos foi de 7,38 milhares/mm^3^ e a das plaquetas foi de 312 milhares/mm^3^. A Razão de Neutrófilos e Linfócitos (RNL) foi de 2,91 milhares/mm^3^, sendo a maioria dos participantes (n=62; 76,54%) com classificação baixa. A média da Razão Plaquetas Linfócitos (RPL) foi de 175,85 milhares/mm^3^ (DP= 90,86) com a maioria (n=59; 72,84%) apresentando baixa RPL. Em relação aos biomarcadores, a média da Troponina I foi de 3,23 pg/mL, sendo que em apenas 1 participante estava elevado, enquanto a Proteína C - reativa (PCR) a média foi de 6,83 mg/dL com 30 participantes apresentando alto risco cardiovascular.

**Tabela 2-:** Hemograma, RNL*, RPL^†^, PCR^‡^ e troponina dos pacientes oncológicos indicados à quimioterapia ambulatorial (n = 84). Vitória, ES, Brasil, 2022-2023

**Variável**	**n**	**%**
**Hemácias (milhões/mm** ^3^ **)**		
Média (Desvio-Padrão)	4,30 (0,56)	-
Mediana (Int. Interquartílico)	4,27 (3,94 - 4,60)	-
**Hemoglobina (g/dL)**		
Média (Desvio-Padrão)	12,53 (1,69)	-
Mediana (Int. Interquartílico)	12,60 (11,60 - 13,50)	-
**Hematócrito (%)**		
Média (Desvio-Padrão)	37,47 (4,67)	-
Mediana (Int. Interquartílico)	37,40 (35,30 - 40,50)	-
**Leucócitos (milhões/mm** ^3^ **)**		
Média (Desvio-Padrão)	7,38 (2,57)	-
Mediana (Int. Interquartílico)	6,95 (5,78 - 8,25)	-
**Plaquetas (milhões/mm** ^3^ **)**		
Média (Desvio-Padrão)	312,00 (108,47)	-
Mediana (Int. Interquartílico)	294,00 (237,00 - 365,00)	-
RNL* (milhões/mm ^3^ **)**		
Média (Desvio-Padrão)	2,91 (3,71)	-
Mediana (Int. Interquartílico)	1,96 (1,57 - 2,66)	-
RNL* (milhões/mm ^3^ **)**		
Baixo	62	76,54
Alto	19	23,46
**RPL** ^†^ **(milhões/mm** ^3^ **)**		
Média (Desvio-Padrão)	175,81 (90,86)	-
Mediana (Int. Interquartílico)	147,92 (115,90 - 202,31)	-
**RPL** ^†^ **(milhões/mm** ^3^ **)**		
Baixo	59	72,84
Alto	22	27,16
**Troponina I (pg/mL)**		
Média (Desvio-Padrão)	3,23 (5,44)	-
Mediana (Int. Interquartílico)	1,50 (0,80 - 2,83)	-
**Troponina I (pg/mL)**		
Normal	83	98,81
Elevado	01	1,19
**PCR** ^‡^ **(mg/dL)**		
Média (Desvio-Padrão)	6,83 (18,84)	-
Mediana (Int. Interquartílico)	1,67 (0,68 - 4,41)	-
**PCR** ^‡^ **(mg/dL) (risco cardiovascular)**		
Baixo risco	30	35,71
Risco moderado	24	28,57
Alto risco	30	35,71

*RNL = Razão Neutrófilos/Linfócitos; ^†^RPL = Razão Plaquetas/Linfócitos; ^‡^PCR = Proteína C-Reativa

Na Tabela 3, em relação à classe dos quimioterápicos, observou-se que a combinação de agente taxano, agente alquilante, antraciclina e anticorpo monoclonal foi a mais frequente entre os pacientes sem prolongamento do QTc (n=34) e também entre aqueles com QTc prolongado (n=4). A combinação de antimetabólito e derivado de platina foi utilizada em dois pacientes sem prolongamento e em um com prolongamento, enquanto a combinação de derivado de platina e agente taxano foi utilizada em 11 pacientes sem prolongamento e um com. As demais combinações apresentaram frequências menores. Não houve associação estatisticamente significativa entre as diferentes combinações de quimioterápicos e a ocorrência de prolongamento do QTc (p=0,285).

Quanto aos níveis de PCR, verificou-se que a maioria dos pacientes com QTc prolongado apresentava PCR elevada (n=6), enquanto entre os sem prolongamento, a distribuição foi mais equilibrada entre as categorias: baixo (n=25), moderado (n=20) e alto (n=23). Ainda assim, não foi identificada associação estatisticamente significativa entre o risco cardiovascular estimado pela PCR e a presença de prolongamento do QTc (p=0,329).

Na Tabela 4, a análise da associação do ECG com PCR e variáveis sociodemográficas e clínicas revelou, de maneira geral, ausência de significância estatística para a maioria das variáveis estudadas. No entanto, observou-se associação significativa entre a circunferência da cintura com FC (p=0,037), sendo a taquicardia mais frequente entre pacientes com risco aumentado de adiposidade abdominal, e, ainda, entre presença de onda T invertida com IMC (p=0,005), sendo esse achado mais comum em indivíduos com IMC acima de 20,5, o que pode sugerir relação entre sobrepeso/obesidade e alterações na repolarização ventricular.

**Tabela 3-:** Associação do intervalo QTc* prolongado com a classe do quimioterápico e PCR^†^ (risco cardiovascular) (n = 78). Vitória, ES, Brasil, 2022-2023

**Variáveis**	Intervalo QTc* prolongado		**p** ^‡^
	**Não**	**Sim**	
**Classe do Quimioterápico**			0,285
Antimetabólito + Derivado de Platina	2	1	
Derivado de Platina + Agente Taxano	11	1	
Agente Taxano + Agente Alquilante + Antraciclina + Anticorpo Monoclonal	34	4	
Agente Taxano + Agente Alquilante + Derivado de Platina	1	1	
Antimetabólito + Inibidor da Topoisomerase + Derivado de Platina + Agente Taxano + Análogo do ácido fólico	20	3	
**Proteína C-reativa (risco cardiovascular)**			0,329
Baixo	25	2	
Moderado	20	2	
Alto	23	6	

*QTc = Intervalo QT corrigido; ^†^PCR = Proteína C-reativa; ^‡^p-valor calculado com base no teste exato de Fisher

A Tabela 5 apresenta a associação da história pregressa com ECG e PCR. Houve associação estatisticamente significativa entre o uso de tabaco e o ritmo cardíaco (p = 0,010), com maior frequência de ritmo irregular entre tabagistas. Os demais parâmetros eletrocardiográficos (FC, intervalo QTc, supra de ST, onda T invertida e QRS alargado) não apresentaram associação significativa com as comorbidades avaliadas (p>0,05). Em relação à PCR, observaram-se associações significativas com DM (p = 0,033) e uso de bebida alcoólica (p = 0,033), sendo os níveis elevados mais frequentes entre pessoas com diabetes e consumidores de álcool.

**Tabela 4-:** Associação de parâmetros do ECG^*^dos pacientes oncológicos indicados à quimioterapia ambulatorial com PCR^†^(risco cardiovascular) e variáveis sociodemográficas e clínicas (n = 78). Vitória, ES, Brasil, 2022-2023

**Variáveis**		**Ritmo**				**FC** ^‡^				**Intervalo QTc** ^§^ **prolongado**			**Onda T invertida**				**QRS** ^||^ **alargado**			
	**Regular**	**Irregular**	p**		**Bradicardia**	**Normocardia (60-100)**	**Taquicardia**	p**		**Não**	**Sim**	p**		**Não**	**Sim**	**V1**	**p-valor**		**Não**	**Sim**	p**
**Proteína C-Reativa (risco cardiovascular)**			0,837					0,600			0,329			0,454			0,378
Baixo	26	1			4	23	0			25	2			24	3	0			25	2	
Moderado	20	1			3	18	1			20	2			20	1	1			21	1	
Alto	28	1			3	23	3			23	6			28	1	0			29	0	
**Sexo**			0,066				0,302			0,134				0,727			0,190
Masculino	19	2			4	16	2			17	5			20	2	0			20	2	
Feminino	55	1			6	48	2			51	5			52	3	1			55	1	
**Faixa etária**			0,340				0,701			0,434				0,280			0,471
< 50 anos	19	1			1	18	1			18	2			20	0	0			20	0	
50-64 anos	32	0			4	27	2			30	3			31	2	0			32	1	
≥ 65 anos	23	2			5	19	1			20	5			21	3	0			23	2	
**Cor autorreferida**			1,000				0,884			0,697				0,395			1,000
Branca	29	1			5	24	1			27	3			28	1	1			29	1	
Negro	9	0			2	7	0			7	2			8	1	0			9	0	
Pardo	28	2			3	25	3			27	4			29	2	0			29	2	
Amarelo	3	0			0	3	0			3	0			2	1	0			3	0	
Sem Informação	5	0			0	5	0			4	1			5	0	0			5	0	
**IMC** ^¶^			0,101				1,000			0,241				**0,005**			0,076
Abaixo de 20,5	1	1			0	2	0			1	1			0	2	0			1	1	
Acima de 20,5	73	2			10	62	4			67	9			72	3	1			74	2	
**Circunferência de Cintura**			0,290				**0,037**			0,590				0,712			0,763
Normal	13	2			0	15	0			14	1			13	2	0			14	1	
Risco aumentado	20	0			6	14	0			16	4			19	1	0			20	0	
Risco muito aumentado	41	1			4	35	4			38	5			40	2	1			41	2	
**Circunferência Abdominal**			0,387			0,062			1,000			0,796			0,571
Normal	30	2			7	26	0			29	4			30	3	0			31	2	
Obeso	44	1			3	38	4			39	6			42	2	1			44	1	
**Circunferência Abdominal**			1,000				0,755			0,699			0,151			0,145
Normal	18	1			2	17	0			16	3			16	3	0			17	2	
Obeso	56	2			8	47	4			52	7			56	2	1			58	1	
**Glicemia**			0,425			0,014			0,398			0,450			0,576
Normal	30	1			5	27	0			30	2			30	2	0			30	2	
Pré-Diabético	13	2			2	11	2			13	2			12	2	1			14	1	
Diabético	24	0			0	22	2			19	5			23	1	0			24	0	
Sem informação	7	0			3	4	0			6	1			7	0	0			7	0	
**Estadiamento**			0,738			0,306			0,143			0,199			1,000
I	8	0			2	6	0			7	1			7	1	0			8	0	
II	42	1			6	36	2			37	7			43	1	0			42	2	
III	22	2			2	21	1			23	1			20	3	1			23	1	
Sem Informação	2	0			0	1	1			1	1			2	0	0			2	0	
**Classe do Quimioterápico**			0,118			0,076			0,285			0,381			0,301
Antimetabólito + Derivado de Platina	2	0			2	1	0			2	1			3	0	0			3	0	
Derivado de Platina + Agente Taxano	12	0			3	8	1			11	1			10	2	0			11	1	
Agente Taxano + Agente Alquilante + Antraciclina+ Anticorpo Monoclonal	3	1			2	35	1			34	4			37	1	0			38	0	
Agente Taxano + Agente Alquilante + Derivado de Platina	2	0			0	2	0			1	1			2	0	0			2	0	
Antimetabólito + Inibidor da Topoisomerase + Derivado de Platina + Agente Taxano+ Análogo do ácido fólico	21	2			3	18	2			20	3			20	2	1			21	2	

*ECG = Eletrocardiograma; **†**PCR = Proteína C-reativa; ^‡^FC = Frequência cardíaca;^§^QTc = Intervalo QT corrigido; ^||^QRS = Complexo QRS; ^¶^IMC = Índice de Massa Corporal; **p = p-valor calculado com base no teste exato de Fisher

**Tabela 5-:** Associação da história pregressa dos pacientes oncológicos indicados à quimioterapia ambulatorial com ECG* e Proteína C-reativa (risco cardiovascular) (n = 78). Vitória, ES, Brasil, 2022-2023

**Variáveis**		**HAS** ^†^				**IAM** ^‡^				**Dislipidemia**				**Diabetes mellitus**				**AVE** ^§^				**Doença valvar**				**Faz uso de tabaco**				**Faz uso de bebida alcoólica**				**História familiar de cardiopatia**		
		**Não**	**Sim**	**p** ^||^		**Não**	**Sim**	**p** ^||^		**Não**	**Sim**	**p** ^||^		**Não**	**Sim**	**p** ^||^		**Não**	**Sim**	**p** ^||^		**Não**	**Sim**	**p** ^||^		**Não**	**Sim**	**p** ^||^		**Não**	**Sim**	**p** ^||^		**Não**	**Sim**	**p** ^||^
**Ritmo**				0,793				1,000				1,000				0,582				1,000				1,000				**0,010**				1,000				1,000
Regular	39	35			70	4			65	9			60	14			71	3			73	1			70	3			63	10			25	48	
Irregular	1	2			3	0			3	0			2	1			3	0			3	0			1	1			2	0			1	2	
** **	Sem informação	1	0			1	0			1	0			1	0			1	0			1	0			0	1			1	0			0	1	
**FC** ^¶^				0,343				1,000				1,000				0,328				1,000				1,000				0,690				0,628				0,188
Bradicardia	7	3			10	0			9	1			8	2			10	0			10	0			9	1			9	1			1	9	
Normocardia (60-100)	33	31			60	4			56	8			53	11			61	3			63	1			58	4			54	8			23	40	
** **	Taquicardia	1	3			4	0			4	0			2	2			4	0			4	0			4	0			3	1			2	2	
**Intervalo QTc** ** **prolongado**				0,504				0,429				0,324				0,093				0,341				1,000				0,477				0,337				0,151
Não	37	31			65	3			61	7			57	11			66	2			67	1			63	4			59	8			25	42	
** **	Sim	4	6			9	1			8	2			6	4			9	1			10	0			8	1			7	2			1	9	
**Supra de ST** ^††^				0,474				1,000				1,000				1,000				1,000				1,000				1,000				1,000				1,000
Não	41	36			73	4			68	9			62	15			74	3			76	1			70	5			65	10			26	50	
** **	Sim	0	1			1	0			1	0			1	0			1	0			1	0			1	0			1	0			0	1	
**Onda T invertida**				0,114				1,000				0,533				0,658				0,216				1,000				1,000				1,000				0,214
Não	40	32			68	4			64	8			57	15			70	2			71	1			66	5			61	10			26	45	
Sim	1	4			5	0			4	1			5	0			4	1			5	0			4	0			4	0			0	5	
** **	V1	0	1			1	0			1	0			1	0			1	0			1	0			1	0			1	0			0	1	
**QRS** ^‡‡^ **alargado**				0,601				0,148				1,000				1,000				0,112				1,000				1,000				1,000				0,547
Não	40	35			72	3			66	9			60	15			73	2			74	1			69	5			64	10			26	48	
	Sim	1	2			2	1			3	0			3	0			2	1			3	0			2	0			2	0			0	3	
**Proteína C-Reativa (risco cardiovascular)**				0,247				0,674				0,741				**0,033**				0,774				1,000				0,431				**0,033**				0,688
Baixo	13	14			26	1			25	2			25	2			26	1			27	0			24	1			25	0			9	18	
Moderado	15	7			20	2			19	3			19	3			22	0			22	0			19	3			18	4			6	16	
** **	Alto	13	16			28	1			25	4			19	10			27	2			28	1			28	1			23	6			11	17	

*ECG = Eletrocardiograma; **†**HAS = Hipertensão Arterial Sistêmica; ^‡^IAM = Infarto Agudo do Miocárdio; ^§^AVE = Acidente Vascular Encefálico; ^||^p = p-valor calculado com base no teste exato de Fisher; ^¶^FC = Frequência cardíaca; **QTc = Intervalo QT corrigido; ^††^ST = Segmento ST; ^‡‡^QRS = Complexo QRS

## Discussão

No presente estudo, a maioria dos participantes eram mulheres, com maior incidência de diagnóstico de câncer de mama, seguido de câncer de cólon e brônquios e pulmões. Nessa perspectiva, a evidência corrobora que o câncer de mama é o tumor que mais acomete mulheres no mundo todo, quando se exclui o câncer de pele não melanoma^(31)^. Entretanto, essa distribuição difere das estimativas internacionais^(32)^ e nacional^(33)^, o que pode estar associado aos fatores socioeconômicos, ambientais e outras questões relacionadas ao perfil da população do estudo. À vista disso, um estudo demonstra que os casos de neoplasias malignas de mama têm crescido progressivamente com o avanço da industrialização e urbanização^(34)^.

A média de idade (57,68) está em consonância com o perfil etário comumente descrito para pacientes com câncer^(33-34)^. O envelhecimento, por si só, é um fator de risco para a carcinogênese, sendo associado a comorbidades e à instabilidade genômica, como mutação na telomerase. Portanto, a idade biológica está relacionada a um risco aumentado para qualquer tipo de câncer^(35)^.

No que diz respeito à terapia, verificou-se prevalência do esquema quimioterápico composto por taxanos, alquilantes, antraciclinas e anticorpos monoclonais, comumente recomendado para o tratamento de tumores sólidos, como o câncer de mama, bexiga e pulmão. Essas classes de quimioterápicos são reconhecidas como um dos principais agentes com alto potencial cardiotóxico^(7,36)^.

Um estudo com pacientes com câncer de mama inicial demonstra que os efeitos cardiotóxicos da doxorrubicina (DOX) podem surgir de forma tardia, anos após a exposição à quimioterapia, destacando a importância da identificação precoce dos indivíduos em risco^(7,37)^. Além das antraciclinas, Docetaxel e Ciclofosfamida também têm seus efeitos tóxicos sobre o miocárdio, embora seus mecanismos ainda não estejam totalmente esclarecidos. Ambos estão associados ao risco de disfunção ventricular esquerda (DVE)^(38-40)^, a qual representa a principal manifestação cardiotóxica^(41)^.

Na pesquisa, 23,46% (n=19) e 27,16% (n=22) dos pacientes apresentavam valores basais elevados de RNL e RPL, respectivamente. Contrariamente, um estudo com pacientes com diagnóstico de câncer de mama em tratamento com antraciclina não identificou alteração nos valores basais de RNL, entretanto, observou-se aumento deste marcador após a exposição à quimioterapia, associado a um risco quatro vezes maior para desenvolver disfunção cardíaca, sugerindo maior influência da antraciclina do que inflamação basal causada pela doença^(40)^.

Outro estudo, que relacionou RNL e RPL com o estado nutricional, fatores clínicos e sociodemográficos e qualidade de vida de mulheres hospitalizadas com câncer de mama não metastático, verificou que existe um alto risco para as pessoas com valores médios e elevados de RNL e RPL, o que significa uma baixa atividade antitumoral e um pior prognóstico^(14)^. Esses biomarcadores, relacionados à inflamação sistêmica, vêm sendo estudados como ferramentas acessíveis de estratificação de risco^(41)^.

A PCR, também considerada um importante marcador inflamatório, esteve elevada em 35,71% dos pacientes (n=30), indicando risco cardiovascular aumentado. Em adição, uma revisão sistemática mostrou que a PCR, junto à troponina, peptídeo natriurético tipo B (BNP), está relacionada aos graves desfechos cardiovasculares adversos, como o infarto do miocárdio e a insuficiência cardíaca^(42)^. Neste estudo, observou-se associação entre DM e PCR, indicando maior presença de processo inflamatório nos pacientes. Essa condição contribui para o aumento do risco cardiovascular, bem como para a progressão de complicações vasculares^(43)^.

A monitorização da cardiotoxicidade e inflamação sistêmica por meio da dosagem de biomarcadores séricos, como RNL, RPL, Troponina I e PCR, é um método acessível, minimamente invasivo e de baixo custo que contribui para a estratificação de risco e detecção precoce dessa condição previamente à terapia inicial, bem como contribui para diagnóstico precoce de DCV tanto durante quanto após o tratamento, fornecendo subsídios para detectar pacientes oncológicos que podem ser favorecidos com tratamentos cardioprotetores ou que necessitam de acompanhamento a longo prazo^(44-46)^.

Múltiplos fatores de risco cardiovascular pré-existentes – como hipertensão, diabetes mellitus, dislipidemias e obesidade – são amplamente reconhecidos na literatura como condições que aumentam o risco de toxicidade cardiovascular associada ao tratamento oncológico^(36)^. No entanto, na presente amostra, não houve associações estatisticamente significativas entre variáveis clínicas e laboratoriais e a maioria dos parâmetros eletrocardiográficos avaliados. Apesar disso, destaca-se a identificação de relação entre IMC e onda T invertida (p=0,005). Tal achado é consistente com estudos que relatam o impacto do IMC elevado sobre a função elétrica do miocárdio, favorecendo alterações na repolarização^(47-48)^.

Ainda que o prolongamento do QTc não tenha se associado de forma significativa às variáveis investigadas, observou-se que 10 pacientes já apresentavam essa alteração no momento basal, reforçando a necessidade de monitoramento contínuo. Evidências mostram que o prolongamento do QTc pode ser exacerbado ao longo do tratamento, especialmente com o uso cumulativo de antraciclinas e taxanos, aumentando o risco de arritmias graves^(4,49)^.

A fibrilação atrial, os batimentos ventriculares ectópicos e o próprio prolongamento do QTc estão entre as arritmias mais frequentes em pacientes oncológicos, podendo ser agravadas durante o tratamento^(50-52)^. Nesse contexto, a avaliação eletrocardiográfica deve ser sistematicamente incorporada desde a fase inicial do tratamento.

É importante considerar que a ausência de associações experienciadas neste estudo pode estar relacionada ao fato de os pacientes se encontrarem na fase pré-quimioterapia, período em que os efeitos cardiotóxicos associados ao tratamento ainda não se manifestaram. Assim, embora não haja associações significativas no momento basal, é importante destacar que esses fatores são bem estabelecidos na literatura como preditores de maior risco para o desenvolvimento de cardiotoxicidade, especialmente em pacientes expostos a agentes como as antraciclinas^(7,36)^.

De fato, estudos prévios demonstram que indivíduos com essas comorbidades apresentam até 12 vezes mais chances de desenvolver disfunção cardíaca induzida por antraciclinas^(53)^. Além disso, segundo as Diretrizes de Prática Clínica da Sociedade Americana de Oncologia Clínica, a presença de múltiplos fatores de risco cardiovascular, associada ao uso de antraciclinas e à idade superior a 60 anos, configura um cenário de risco elevado para o desenvolvimento de disfunção cardíaca^(54-55)^. Dessa forma, esta análise comprova ainda mais o impacto das comorbidades sobre o risco cardiotóxico e aponta para a importância do controle das doenças de base, além da avaliação e acompanhamento dos pacientes pela equipe multidisciplinar voltada para o sistema cardiovascular do início ao pós-tratamento quimioterápico como forma de prevenção, seguindo as recomendações da Diretriz Brasileira de Cardio-Oncologia^(36)^.

Também é importante explorar o potencial do ECG e dos biomarcadores séricos, como RNL, RPL, troponina I e PCR, como ferramentas diagnósticas e de monitoramento de alterações cardíacas, bem como para a estratificação de risco cardiovascular, visando à identificação precoce de cardiotoxicidade e à seleção de pacientes que possam se beneficiar de intervenções cardioprotetoras^(41)^.

Embora os biomarcadores inflamatórios e cardíacos analisados neste estudo possuam reconhecida relevância clínica, é importante considerar que a avaliação foi realizada na fase pré-quimioterapia, período anterior à manifestação plena dos efeitos citotóxicos e inflamatórios mais intensos decorrentes do tratamento antineoplásico. Isso pode justificar a ausência de associações estatisticamente significativas entre esses marcadores e os parâmetros clínicos ou eletrocardiográficos avaliados. A baixa elevação da troponina I, observada em apenas um paciente, limita sua aplicabilidade como marcador basal, embora sua utilidade prognóstica esteja bem estabelecida em fases subsequentes do tratamento, especialmente como preditor de disfunção ventricular induzida por antraciclinas^(8,28)^. Do mesmo modo, a PCR elevada, identificada em 35,7% da amostra, quando utilizada isoladamente, apresenta baixa especificidade como marcador de risco cardiovascular, podendo ser influenciada por outras condições inflamatórias, infecciosas ou metabólicas, como o estado nutricional, presença de diabetes mellitus ou carga tumoral^(15,42)^. Ainda assim, a detecção precoce desses biomarcadores, mesmo em níveis subclínicos, pode contribuir para a estratificação individualizada de risco e orientar estratégias preventivas em cardio-oncologia, especialmente em pacientes com comorbidades ou fatores de risco modificáveis. Tal abordagem encontra respaldo nas diretrizes internacionais e nas evidências mais recentes sobre biomarcadores na monitorização da cardiotoxicidade^(41,54)^.

Os profissionais de saúde, especialmente os enfermeiros, desempenham um papel fundamental na vigilância clínica de pacientes oncológicos, sendo imprescindível que estejam atentos ao monitoramento e à avaliação de biomarcadores séricos e do ECG para a identificação precoce e estratificação dos riscos cardiovasculares, tanto durante o tratamento quanto no acompanhamento (*follow-up*). Diante da reconhecida cardiotoxicidade de diversos agentes quimioterápicos, a intervenção oportuna pode minimizar complicações e melhorar o prognóstico^(7,36)^.

Nesse contexto, destaca-se a crescente incorporação das ciências ômicas à prática clínica da Enfermagem^(56-58)^, reforçando a importância da Enfermagem de Precisão^(51-52)^. A aplicação de estratégias personalizadas, baseadas em biomarcadores específicos e nas características individuais de cada paciente contribui para um cuidado mais eficaz e personalizado, centrado no indivíduo e orientado para a tomada de decisão clínica baseada em evidências^(58-60)^.

Portanto, é imprescindível a adoção de protocolos de monitoramento, aliados a intervenções terapêuticas fundamentadas em evidências, com o objetivo de mitigar a cardiotoxicidade, reduzir a morbidade e mortalidade cardiovascular e, consequentemente, melhorar o prognóstico dos pacientes oncológicos^(60)^. As diretrizes internacionais como as da *European Society of Cardiology* em conjunto com a *European Society for Medical Oncology* (ESMO), bem como da *American Society of Clinical Oncology* (ASCO), reforçam a necessidade de acompanhamento estruturado e sistemático para pacientes expostos a agentes cardiotóxicos. A adoção dessas recomendações possibilita a detecção precoce de disfunção miocárdica subclínica, permitindo intervenções oportunas e efetivas para prevenir a progressão para insuficiência cardíaca e outros eventos cardiovasculares adversos^(7)^.

Os achados deste estudo possuem implicações relevantes para a prática clínica da Enfermagem, especialmente no contexto da Enfermagem Oncológica. A identificação de biomarcadores inflamatórios e cardíacos ainda na fase pré-quimioterapia, mesmo em níveis subclínicos, evidencia a necessidade de uma atuação proativa da equipe de Enfermagem na estratificação precoce de risco cardiovascular^(60-61)^. Marcadores como RNL, RPL, troponina I e PCR, por serem acessíveis e de baixo custo, podem ser incorporados como ferramentas complementares à anamnese e ao exame físico na triagem cardiovascular, subsidiando o planejamento de intervenções personalizadas e vigilância contínua ao longo do tratamento oncológico. A realização sistemática de exames como o ECG, associada à interpretação crítica dos biomarcadores, fortalece o papel da Enfermagem na detecção precoce de alterações cardiovasculares, na comunicação com a equipe multiprofissional e na adoção de estratégias cardioprotetoras.

Ademais, a integração dessas práticas ao protocolo de avaliação inicial do paciente oncológico favorece o cuidado integral, seguro e baseado em evidências, alinhado aos princípios da Enfermagem de Precisão e às diretrizes de cardio-oncologia nacionais e internacionais. Tais evidências reforçam a importância da enfermagem como protagonista na triagem, monitoramento e educação em saúde cardiovascular no cuidado oncológico, contribuindo diretamente para a prevenção de agravos e para a melhoria dos desfechos clínicos desses pacientes.

As limitações deste estudo incluem: I. A elevação discreta da troponina I, detectada em apenas um paciente, o que limita a interpretação da aplicabilidade deste marcador em condições basais; II. O estudo foi conduzido em um único centro especializado no tratamento do câncer, com uma amostra relativamente pequena e heterogênea, o que pode comprometer a validade externa e a generalização dos achados para outras populações ou contextos clínicos; III. Apesar de o número total de pacientes ter alcançado a amostra mínima estimada (n=84), algumas análises específicas apresentaram perdas amostrais pontuais devido à inconsistência ou ausência de dados laboratoriais e/ou eletrocardiográficos, o que reduziu o número de participantes em determinadas variáveis e pode ter impactado a potência estatística dessas análises; IV. A ausência de grupo controle composto por pacientes oncológicos não submetidos à quimioterapia ou sem fatores de risco cardiovascular; V. O delineamento transversal do estudo, que impossibilita o acompanhamento longitudinal das alterações inflamatórias e eletrocardiográficas ao longo do tratamento oncológico; VI. A não realização de exames complementares importantes, como a dosagem de NT-ProBNP e a ecocardiografia, que poderiam contribuir para uma avaliação mais abrangente da função cardíaca; VII. A utilização da PCR como marcador isolado na estratificação de risco cardiovascular, o que limita sua aplicabilidade clínica, sobretudo na ausência de outros parâmetros complementares.

No entanto, apesar das limitações, nosso estudo apresenta aspectos relevantes, tais como: I. Avaliação de medidas subjetivas e objetivas (por meio de dosagem de biomarcadores); II. Coletas padronizadas de biomarcadores e dados clínicos, antes da infusão da primeira sessão de quimioterapia, garantindo a confiabilidade e validade dos dados; III. Relevância clínica dos resultados para estratificação de risco cardiovascular precoce, com vistas ao bem-estar e ao melhor prognóstico de pacientes oncológicos em quimioterapia. Reforçamos, contudo, que os dados apresentados oferecem importantes evidências preliminares sobre a avaliação cardiovascular basal em pacientes oncológicos na fase pré-quimioterapia, contribuindo para fundamentar futuras investigações multicêntricas e longitudinais.

Propõe-se a condução de estudos longitudinais com amostra representativa e bem delineados para compreender as tendências ao longo do tempo nos diagnósticos de câncer e identificar os fatores de risco cardíacos associados no *follow-up*. Além disso, sugere-se a investigação de estratégias de prevenção e intervenção destinadas a mitigar o risco de prejuízo cardíaco decorrente do tratamento quimioterápico, com foco em cardio-oncologia e Enfermagem de Precisão.

## Conclusão

No presente estudo, a ausência de associações estatisticamente significativas entre a maioria dos parâmetros avaliados pode estar relacionada ao fato de os pacientes se encontrarem na fase pré-quimioterapia, período anterior ao início dos efeitos cardiotóxicos esperados. Ainda assim, foi possível identificar fatores de risco cardiovascular relevantes, como inflamação sistêmica e IMC elevado, os quais, em conjunto com determinados protocolos quimioterápicos, podem potencializar o risco de disfunção cardíaca.

Esses achados reforçam a necessidade de incorporar rotinas de triagem cardiovascular sistemáticas antes da primeira infusão quimioterápica, portanto, desde o início do seguimento oncológico, como estratégia essencial para a detecção precoce de alterações cardiovasculares e para a implementação de intervenções preventivas, personalizadas e em tempo oportuno.

## Data Availability

Todos os dados gerados ou analisados durante este estudo estão incluídos neste artigo publicado.

# Translation: Cardiotoxicidad inducida por quimioterapia en pacientes con cáncer: estudio observacional en un hospital de referencia oncológica en el sudeste de Brasil*

**DOI:** 10.1590/1518-8345.7472.4773

**Authors:** Karoline Neumann, Karolini Zuqui Nunes, Wesley Rocha Grippa, Naira Santos D’Agostini, Sara Isabel Pimentel de Carvalho Schuab, Luís Carlos Lopes-Júnior

**Affiliations:** 1 Universidade Federal do Espírito Santo, Vitória, ES, Brasil. Universidade Federal do Espírito Santo ES Vitória Brasil; 2 Becaria de la Fundação de Amparo à Pesquisa e Inovação do Espírito Santo (FAPES), Brasil. Becaria de la Fundação de Amparo à Pesquisa e Inovação do Espírito Santo Brasil; 3 Universidade Federal do Espírito Santo, Departamento de Enfermagem, Vitória, ES, Brasil. Universidade Federal do Espírito Santo Departamento de Enfermagem ES Vitória Brasil; 4 Becario del Conselho Nacional de Desenvolvimento Científico e Tecnológico (CNPq), Brasil. Becario del Conselho Nacional de Desenvolvimento Científico e Tecnológico Brasil

**Keywords:** Neoplasias, Antineoplásicos, Factores de Riesgo de Enfermedad Cardiaca, Biomarcadores, Electrocardiograma, Cardiotoxicidade

## Abstract

evaluar el perfil clínico, inflamatorio y electrocardiográfico de pacientes con cáncer en la fase pre-quimioterapia, con el objetivo de identificar tempranamente signos indicativos de toxicidad cardiovascular.

estudio observacional, transversal, realizado entre noviembre de 2022 y diciembre de 2023, con adultos con cáncer en estadios I a III, tratados en un hospital del sudeste de Brasil. Se recolectaron datos sociodemográficos, clínicos y tumorales, además de pruebas de laboratorio y electrocardiográficas realizadas antes de la primera infusión de quimioterapia. Se realizaron análisis descriptivos utilizando el *software* R.

participaron 84 pacientes, la mayoría mujeres (72,6%) con diagnóstico de cáncer de mama (57,1%). Hubo una prevalencia de inflamación sistémica, con proteína C reactiva elevada en 35,7%, una relación neutrófilos-linfocitos aumentada en 23,5% y una relación plaquetas-linfocitos en 27,2%. Se observó una asociación significativa entre el índice de masa corporal elevado y las alteraciones de la onda T (p=0,005). Aunque 10 pacientes presentaron un intervalo QT corregido prolongado, no se identificaron otras asociaciones estadísticamente significativas.

la fase pre-quimioterapia reveló alteraciones inflamatorias y electrocardiográficas relevantes, incluso en ausencia de cardiotoxicidad establecida. Estos hallazgos refuerzan la importancia de la evaluación cardiovascular basal y la monitorización multidisciplinaria en cardiología oncológica, especialmente en pacientes con factores de riesgo modificables. Se recomiendan estudios longitudinales para dilucidar las trayectorias clínicas y pronósticas de estas alteraciones.

## Destacados

**(1)** Los pacientes con antraciclinas y taxanos tienen un mayor riesgo de cardiotoxicidad. **(2)** La proteína C reactiva aumentó en la primera infusión de quimioterapia. **(3)** Los índices neutrófilo-linfocito (INL) y plaqueta-linfocito (IPL) son elevados en hasta el 27% de los pacientes antes del inicio del tratamiento. **(4)** La estratificación temprana del riesgo permite el manejo de la cardiotoxicidad. **(5)** El uso de biomarcadores amplía el potencial de la Enfermería de Precisión en la atención personalizada.

## Introducción

A nivel mundial, las enfermedades no transmisibles (ENT) son las principales causas de morbilidad y mortalidad de la población. Esto se debe a la transición demográfica que contribuye al envejecimiento poblacional, junto con la transición epidemiológica marcada por patrones cambiantes de enfermedad y mortalidad, y se considera un importante desafío mundial^(1)^. Entre las ENT, las enfermedades crónicas no transmisibles (ECNT) se destacan en términos de mortalidad a nivel mundial, siendo las enfermedades cardiovasculares (ECV) la principal causa de muerte, al representar casi 18 millones de muertes^(2)^, seguidas de las neoplasias, que representan casi 10 millones de muertes. En la mayoría de los países, el cáncer es la primera o segunda causa principal de muerte antes de los 70 años^(3)^.

En los últimos años, se han producido avances significativos en el tratamiento del cáncer gracias al descubrimiento de nuevos fármacos y terapias que mejoran el pronóstico de los pacientes oncológicos^(4-5)^. Sin embargo, a pesar de los beneficios, las enfermedades cardiovasculares se han convertido en uno de los principales efectos adversos de la terapia antineoplásica, contribuyendo a la morbilidad y mortalidad de los pacientes^(4-6)^. Si bien los agentes quimioterapéuticos desempeñan un papel importante en el pronóstico de estos pacientes, el uso de algunas clases de agentes quimioterapéuticos ha provocado un aumento de los casos de efectos secundarios y complicaciones cardiovasculares, siendo el desarrollo de cardiotoxicidad inducida por agentes quimioterapéuticos la ocurrencia más común, que se extiende después del tratamiento^(6)^.

La Sociedad Europea de Cardiología (*European Society of Cardiology*, ESC) define la toxicidad cardiovascular (TCV) como una lesión cardíaca tanto funcional cuanto estructural condicionada por el tratamiento oncológico (quimioterapia, radioterapia o el propio cáncer)^(7-8)^. Además, la TCV puede dividirse en aguda, subaguda o tardía, en la que los signos y síntomas de esta toxicidad pueden aparecer varios años después del tratamiento del paciente oncológico, afectando las estructuras cardíacas y generando insuficiencia cardíaca (IC), enfermedad coronaria, valvulopatía, arritmias, enfermedad de la conducción cardíaca y enfermedad pericárdica^(8)^.

La cardiotoxicidad, en un inicio, significaba caída de la fracción de eyección, sin embargo, la I Directriz Brasileña de Cardio-Oncología de la Sociedad Brasileña de Cardiología redefinió esta condición como: miocardiopatía con fracción de eyección del ventrículo izquierdo (FEVI) disminuida; síntomas de IC; signos condicionados por la IC; reducción de la FEVI de 5% a menos de 55% cuando se compara con el valor basal y presentar signos o síntomas de IC, o disminución de 10% de la FEVI a menos de 55% sin signos o síntomas simultáneos^(9-10)^.

La aparición de toxicidad cardíaca durante el tratamiento del cáncer puede limitar el tratamiento, lo que puede provocar su interrupción o suspensión y empeorar los resultados clínicos de los pacientes^(11)^. El seguimiento de las enfermedades cardíacas y de los factores de riesgo es esencial y puede mejorar el pronóstico del paciente^(7)^.

A pesar de los recientes avances en la comprensión de la cardiotoxicidad inducida por antineoplásicos, la mayoría de los estudios se centran en resultados clínicos tardíos o en poblaciones que ya sometidas a múltiples ciclos de tratamiento, lo que deja una brecha significativa en el perfil cardiovascular basal de los pacientes con cáncer antes del inicio de la quimioterapia. Como señala una revisión sistemática^(12)^, si bien existe amplia documentación sobre la cardiotoxicidad en pacientes con cáncer hematológico, faltan estudios que investiguen los cambios inflamatorios y electrocardiográficos tempranos en poblaciones con neoplasias sólidas, especialmente en Latinoamérica y en los servicios públicos de referencia oncológica.

En este sentido, nuestro estudio se distingue por su originalidad al evaluar marcadores inflamatorios y cambios electrocardiográficos en la fase pre-quimioterapia en pacientes con cáncer en estadios tempranos a intermedios (estadios I a III), contribuyendo a la detección temprana de posibles signos de cardiotoxicidad incluso antes de la exposición acumulada a la terapia antineoplásica. Este es, por lo tanto, un estudio original que busca ampliar la comprensión de la fase basal del recorrido cardio-oncológico, con potencial impacto en la estratificación del riesgo y la planificación de cuidados multiprofesionales. En este contexto, el objetivo de este estudio fue evaluar los perfiles clínicos, inflamatorios y electrocardiográficos de pacientes con cáncer en la fase pre-quimioterapia, con el objetivo de alcanzar la identificación temprana de signos indicativos de toxicidad cardiovascular.

## Método

### Diseño del estudio

Estudio observacional transversal, realizado de conformidad con lo dispuesto por el método STROBE (*Strengthening the Reporting of Observational Studies in Epidemiology*)^(13)^ para garantizar la transparencia y calidad en la presentación de datos y resultados.

### Escenario

Esta investigación se llevó a cabo en Afecc-Hospital Santa Rita de Cássia (HSRC), único Centro de Alta Complejidad en Oncología (CACON) de Espírito Santo, Brasil - referencia en el estado en tratamiento oncológico para todo Espírito Santo, el sur de Bahía, el este de Minas Gerais y el norte de Río de Janeiro^(14)^- de noviembre de 2022 a diciembre de 2023.

### Participantes

Fueron seleccionadas personas de ambos sexos mayores de 18 años con diagnóstico anatomopatológico de neoplasias malignas en estadio I, II o III, independientemente del tipo de tumor, y solo casos nuevos de pacientes sometidos a quimioterapia ambulatoria. Se excluyó a los participantes con enfermedad en estadio IV, a quienes recibían cuidados paliativos exclusivos y a quienes presentaban más de un tumor primario.

### Variables

Los datos sociodemográficos y la caracterización tumoral se obtuvieron exclusivamente a través de las historias clínicas y un cuestionario semiestructurado. Se tomaron muestras de sangre para la evaluación de biomarcadores: hs-PCR y Troponina I (variables de resultado). Los análisis de biomarcadores se realizaron en el Laboratorio Tomasi, ubicado en Afecc-HSRC. Cabe destacar que los hemogramas completos eran rutinarios en el hospital, por lo que la información se extrajo directamente de las historias clínicas de los pacientes. Para la toma de sangre, cada participante donó una muestra de sangre venosa periférica (4 ml), obtenida mediante venopunción en la fosa cubital. Para este fin, se utilizaron tubos de vacío *Vacutainer*^®^*ethyldiamine tetra-acetic acid* (EDTA) [Becton Dickinson (BD)*,* Franklin Lakes, NJ, EE. UU.]. Para la obtención del plasma, las muestras biológicas recolectadas se procesaron siguiendo el siguiente protocolo: centrifugando los tubos *Vacutainer* que contenían sangre durante 10 minutos a 4 C, 581,2 g/2000 rpm (Eppendorf Centrífuga 5810R). Posteriormente, se alicuotó el plasma con una pipeta y se transfirió a microtubos de almacenamiento. Todo el material alicuotado se conservó en el congelador a -80 C hasta la cuantificación de biomarcadores.

Los puntos de corte adoptados para los biomarcadores se definieron con base en guías clínicas y literatura científica actualizada. Para la proteína C reactiva de alta sensibilidad (hs-PCR), se utilizó la clasificación de la *American Heart Association* (AHA), que categoriza el riesgo cardiovascular como: riesgo bajo (<1 mg/dL), riesgo moderado (1–3 mg/dL) y riesgo alto (>3 mg/dL), respaldada por estudios clínicos y de laboratorio que validaron su uso pronóstico^(15)^. La hs-PCR se obtuvo utilizando el ensayo *Multigent Vario Architect*^®️^ (Abbott Laboratories, Abbott Park, IL, EE. UU.), que es un inmunoensayo de látex. El límite inferior de detección es de 0,01 mg/dL. El coeficiente de variación total del ensayo es ≤6%^(16)^. La cuantificación de la proteína troponina I se realizó mediante inmunoensayo. La troponina I se considera el estándar de oro entre los marcadores bioquímicos de necrosis miocárdica, presentando una excelente sensibilidad y especificidad^(17)^. También se utiliza para el diagnóstico de infarto agudo de miocardio, ya que esta prueba bioquímica tiene un alto valor pronóstico en estos casos y también sirve para la estratificación del riesgo en síndromes coronarios agudos. Además, la troponina I sirve para detectar los efectos agudos de la lesión miocárdica relacionados con la toxicidad de la quimioterapia^(18)^. Un consenso del Comité de Expertos para el *Food and Drug Administration*, concluyó que la troponina I (cTnI) y la troponina T (cTnT) son biomarcadores sensibles, específicos y robustos de daño cardíaco y permiten la detección y cuantificación de daño y muerte celular relacionada con la prueba de nuevos fármacos^(18)^. Los valores de referencia para la troponina I cardíaca son, respectivamente: menos de 0,01 mg/L (por el método de ensayo inmunoabsorbente ligado a enzimas). En el caso de la troponina I, el límite inferior de detección del inmunoensayo de electroquimioluminiscencia utilizado en el presente estudio (<0,01 mg/L) se adoptó como el punto de referencia para la normalidad, según lo recomendado por estudios internacionales centrados en la cardiotoxicidad subclínica inducida por quimioterapia^(18)^.

El índice neutrófilo-linfocito (INL) se calculó dividiendo los recuentos absolutos de neutrófilos y linfocitos, y se clasificó como alto cuando ≥3,0 y bajo cuando <3,0, como se demostró en estudios de pacientes con cáncer de mama y otros tumores sólidos^(19-20)^. El índice plaqueta-linfocito (IPL) se definió como alto cuando >200, lo que indica inflamación sistémica persistente y peor actividad antitumoral, como se demostró en investigaciones previas^(21-23)^.

### Fuentes de datos

Inicialmente, se analizaron las agendas de los oncólogos clínicos del Afecc-HSRC para identificar a los posibles pacientes con derivaciones para quimioterapia por primera vez. Tras la consulta con el oncólogo clínico, el paciente fue derivado al departamento de quimioterapia para su programación. En este punto, se contactó al paciente para invitarlo a participar en el estudio, de acuerdo con los criterios de elegibilidad. Durante este acercamiento inicial, se presentaron los objetivos de la investigación y quienes aceptaron firmaron un Formulario de Consentimiento Libre e Informado. La recopilación de datos se llevó a cabo entre noviembre de 2022 y diciembre de 2023 y fue realizada por enfermeros oncológicos y estudiantes de grado de Enfermería y Obstetricia de la *Universidade Federal do Espirito Santo* (UFES), miembros del *Grupo de Estudos e Pesquisa em Oncología*(GEPONC)/*Conselho Nacional de Desenvolvimento Científico e Tecnológico*(CNPq), quienes fueron capacitados y supervisados por los investigadores principales. Participaron en la capacitación estudiantes de enfermería que hubiesen cursado las materias “Semiología y Semiótica” y “Enfermería Oncológica”, impartidas por el investigador principal, así como los enfermeros oncológicos para organizar y guiar a todos los recopiladores de datos. La capacitación consistió en la administración grupal de los cuestionarios e instrumentos del proyecto, así como simulaciones de exámenes físicos y el uso estandarizado y correcto del electrocardiograma. Se invitó a un nutricionista, máster en Nutrición y miembro del GEPONC/CNPq, para realizar las simulaciones de mediciones antropométricas.

Los investigadores principales elaboraron un cuestionario sociodemográfico y clínico basado en una revisión sistemática de la literatura sobre el tema, así como en las variables clínicas y epidemiológicas contenidas en el Formulario de Registro de Tumores del Registro de Cáncer Hospitalario de la Afecc (HSRC). Se accedió a las historias clínicas para obtener información más detallada sobre aspectos clínicos, tratamiento oncológico y datos del hemograma. Además, se realizó una consulta de enfermería que incluyó la recopilación de la historia clínica, la exploración física, la evaluación antropométrica y el electrocardiograma (ECG).

En el análisis del ECG se evaluaron las siguientes variables: duración del complejo QRS e intervalo QT corregido (QTc)^(24)^. Según las Directrices de la Sociedad Brasileña de Cardiología (SBC), un QRS se considera ancho cuando su duración es mayor a 120 milisegundos (0,12 segundos). El intervalo QT corresponde al tiempo entre el inicio del complejo QRS y el final de la onda T, representando la duración total de la actividad eléctrica ventricular. Sin embargo, como el intervalo QT está directamente influenciado por la frecuencia cardíaca, es común utilizar el intervalo QT corregido (QTc), calculado por la fórmula de Bazett. Los valores de referencia para QTc varían según el sexo, considerándose normales hasta 450 milisegundos (ms) para hombres y hasta 470 ms para mujeres^(24)^.

Los datos del cuestionario incluyeron: edad, sexo, raza declarada por el paciente, nivel educativo, estado civil, tiempo transcurrido desde el diagnóstico hasta el inicio del tratamiento (en días), antecedentes y presencia de comorbilidades, tabaquismo, alcoholismo, Clasificación Internacional de Enfermedades (CIE-10) y estadificación. El examen físico incluyó un estudio cardiovascular (frecuencia cardíaca, ritmo cardíaco, amplitud cardíaca, perfusión periférica, distensión yugular, edema de extremidades, *ictus cordis* y auscultación cardíaca) y una evaluación antropométrica (peso, altura, circunferencia abdominal y circunferencia de la cintura).

El examen cardiovascular consistió en inspección, palpación, percusión y auscultación, según lo estandarizado. Se realizó la inspección de la vena yugular para evaluar la presión venosa central e identificar signos de turgencia yugular. La frecuencia cardíaca (FC) se evaluó colocando las yemas de los dedos índice y medio en la región anterolateral de la muñeca y midiendo el número de latidos por minuto del pulso radial. El valor de referencia de la FC adoptado siguió la Guía Brasileña, con un rango entre 50 y 99 lpm^(24)^ considerado normal. La palpación también evaluó la regularidad e irregularidad del ritmo cardíaco y la amplitud, observando cualquier anomalía. El *ictus cordis* se inspeccionó y palpó en el tórax entre el cuarto y quinto espacio intercostal izquierdo, a nivel de la línea medioclavicular izquierda.

La auscultación cardíaca se realizó con la ayuda de un estetoscopio doble (Bic^®^*Eternity*), que permitió identificar ruidos cardíacos normales (S1 o S2) o incluso la detección de ruidos anormales como soplos, chasquidos o murmullos. Para el ECG, se utilizó un electrocardiógrafo (Cardiocare 2000 12-channel Bionet^®^) con 12 derivaciones. Los participantes se tumbaban en la camilla, se quitaban la parte superior de la ropa y el recolector posicionaba los electrodos en el tórax (en las posiciones V1 a V6), las muñecas y los tobillos.

Los datos de la evaluación antropométrica se recogieron por triplicado, y el promedio se calculó utilizando mediciones estandarizadas. Para medir el peso, se utilizó una balanza (Omron^®^) con capacidad para 150 kg y precisión de 0,1 kg, en la que los participantes se colocaron de pie sobre el aparato, descalzos y con la mínima ropa posible. Para medir la altura, los pacientes permanecieron en posición erguida, con los brazos extendidos a lo largo del cuerpo y la mirada fija en el horizonte, y la medición se realizó con un estadiómetro portátil (Sanny^®^) con una precisión de 1 mm. Para medir la circunferencia abdominal (CA) y la circunferencia de la cintura (CC), se utilizó una cinta métrica inextensible e inelástica (Cescorf^®^). La CC se midió desde el punto medio entre la cresta ilíaca y el borde inferior de la última costilla con los individuos en posición erguida, pies juntos, ropa superior levantada y brazos cruzados en la región del pecho. Del mismo modo, la CA se midió a la altura de la cicatriz umbilical.

Es de destacar que todos los biomarcadores y el ECG se recogieron al mismo tiempo para todos los pacientes, es decir, antes de la infusión de la primera sesión de quimioterapia ambulatoria, con el fin de minimizar el sesgo de medición.

### Tamaño de la muestra

Para calcular el tamaño muestral, se consideró la serie de casos del servicio donde se reclutaron los pacientes y de estudios previos realizados con pacientes en el Hospital mencionado^(25-27)^. Por lo tanto, considerando la incidencia estimada de cardiotoxicidad inducida por quimioterapia basada en la literatura científica en torno al 40%^(28-29)^ (p=0,40), se calculó el número de muestra, fijando α en el 5% (error tipo I) y teniendo en cuenta una potencia estadística de prueba del 80% (β=0,20). Además, se añadió un 20% al tamaño muestral en el cálculo considerando la probabilidad de pérdidas/abandonos. La fórmula utilizada para el cálculo de la muestra fue propuesta inicialmente por Kish (1965): n=N.Z^2^.p.(1-p) / Z^2^.p.(1-p) + e^2^.N-1 (n: muestra calculada, N: población, Z: variable normal, p: probabilidad real del evento, e: error de muestreo)^(30)^.

Considerando la población de pacientes diagnosticados (solo casos nuevos) en Afecc-Hospital Santa Rita de Cássia en 2022 (n=3513) sin el sesgo de la pandemia del nuevo coronavirus, y fijando α en 5% (error de muestreo), con nivel de confianza del 95% y porcentaje mínimo del 80% (considerando pérdidas del 20%), el tamaño de la muestra de esta investigación fue de 84 pacientes.

### Análisis de datos

Las variables categóricas se presentaron como frecuencias absolutas y relativas, mientras que las variables numéricas se resumieron utilizando medidas de tendencia central (media y mediana) y dispersión (desviación estándar y rango intercuartil). Se utilizó la prueba exacta de Fisher para el análisis inferencial para evaluar posibles asociaciones entre parámetros electrocardiográficos, biomarcadores inflamatorios y características clínicas y sociodemográficas de los pacientes. El análisis de datos incluyó un total de 84 participantes, de acuerdo con el tamaño mínimo de muestra estimado previamente. Sin embargo, algunos análisis inferenciales específicos mostraron pequeñas variaciones muestrales (n<84) debido a información faltante ocasional en ciertas pruebas de laboratorio o electrocardiográficas. Estos datos faltantes se debieron principalmente a errores técnicos en el procesamiento de las muestras, registro inconsistente o no disponibilidad de la recolección de muestras el mismo día del cribado clínico. Se consideró un nivel de significancia del 5% (p<0,05). Todos los análisis se realizaron utilizando el *software* R (versión 4.3.2) en el entorno *RStudio* (versión 2023.09.1 Build 494).

### Aspectos éticos

El estudio fue aprobado por el Comité de Ética en Investigación con CAAE: 56492222.6.0000.5060 y Dictamen No. 5,310,994. Se obtuvo también el permiso de la institución hospitalaria en los sectores pertinentes para la recolección de datos. Los participantes, tras expresar voluntariamente su deseo de participar en el estudio, firmaron el Formulario de Consentimiento Libre e Informado. Cabe señalar que el anonimato de la información de los participantes fue protegido mediante el cifrado de los datos en la base de datos, la eliminación de identificadores personales y la restricción del acceso a la base de datos a tres investigadores que conformaron el equipo de investigación, con el fin de priorizar los principios de confidencialidad y privacidad relacionados con el presente estudio.

## Resultados

Se incluyó a 84 pacientes con diagnóstico de cáncer, con media de edad de 57,6 años y desviación estándar de 11,8 años. La mayoría de los participantes eran mujeres (n=61; 72,62%), mestizas (n=34; 40,48%) y casadas (n=43; 51,19%). En cuanto al nivel de estudios, el 38,10% (n=32) había completado la educación primaria y el 30,95% (n=26) tenía educación secundaria incompleta.

El diagnóstico de neoplasia maligna de mama representó el 57,14% (n=48), seguido por neoplasia de colon con el 22,62% (n=19) y neoplasia maligna de bronquios y pulmones con el 5,95% (n=5). En cuanto a la estadificación clínica del tumor, el tipo II fue el más prevalente, presente en el 57,14% (n=48) de los participantes, seguido por el tipo III (n=24; 28,57%) y el tipo I (n=9; 10,71%). Las características demográficas y clínicas de los pacientes se presentan en la Tabla 1.

**Tabla 1-:** Caracterización sociodemográfica y clínica de los pacientes con cáncer remitidos a quimioterapia ambulatoria (n = 84). Vitória, ES, Brasil, 2022-2023

**Variables**	**n**	**%**
**Edad (en años)**		
Media (desviación estándar)	57,68 (11,81)	-
Mediana (rango intercuartil)	58,00 (49,75 - 67,00)	-
**Rango de edad**		
<50 años	21	25,00
50-64 años	36	42,86
≥65 anos	27	32,14
**Sexo**		
Masculino	23	27,38
Femenino	61	72,62
**Color de piel autoinformado**		
Blanco	32	38,10
Negro	10	11,90
Marrón	34	40,48
Amarillo	3	3,57
Sin información	5	5,95
**Educación**		
Analfabeto	5	5,95
Educación primaria incompleta	9	10,71
Educación primaria completa	32	38,10
Educación secundaria incompleta	26	30,95
Educación secundaria completa	3	3,57
Educación superior incompleta	1	1,19
Educación superior completa	4	4,76
Estudios de posgrado	4	4,76
**Estado civil**		
Soltero	15	17,86
Casado	43	51,19
Viudo	11	13,10
Divorciado	11	13,10
Unión estable	4	4,76
CIE-10*		
C18 - Neoplasia maligna de colon	19	22,62
C50 - Neoplasia maligna de mama	48	57,14
C54.1 - Neoplasia maligna del endometrio	1	1,19
C67 - Neoplasia maligna de vejiga	2	2,38
C60 - Neoplasia maligna de pene	1	1,19
C34 - Neoplasia maligna de bronquios y pulmones	5	5,95
C31.9 - Neoplasia maligna de los senos paranasales	1	1,19
C160 - Neoplasia del cardias	1	1,19
C44 - Neoplasia maligna indiferenciada	1	1,19
C22.1 - Colangiocarcinoma	1	1,19
C56 - Neoplasia maligna de ovario	1	1,19
C16 - Neoplasia maligna de estómago	1	1,19
C48 - Neoplasia maligna de los tejidos blandos del retroperitoneo y del peritoneo	1	1,19
C15 - Neoplasia maligna del esófago	1	1,19
**Tratamiento**		
Quimioterapia	48	57,14
Cirugía + quimioterapia	26	30,95
Quimioterapia + terapia hormonal y radioterapia	1	1,19
Quimioterapia + radioterapia	3	3,57
Quimioterapia + cirugía	6	7,14
**Estadificación**		
I	9	10,71
II	48	57,14
III	24	28,57
Sin información	3	3,57

*CIE-10 = Clasificación Internacional de Enfermedades y Problemas Relacionados con la Salud-10

En la Tabla 2 se presenta la información obtenida a partir del hemograma y los biomarcadores de pacientes oncológicos. En cuanto a la serie roja, el recuento medio de glóbulos rojos fue de 4,3 millones/mm^3^, la hemoglobina de 12,53 g/dL y el hematocrito de 37,47%. El recuento medio de leucocitos fue de 7,38 mil/mm^3^ y el de plaquetas de 312 mil/mm^3^. El índice neutrófilo-linfocito (INL) fue de 2,91 mil/mm^3^ y la mayoría de los participantes (n=62; 76,54%) presentó una clasificación baja. El índice plaqueta-linfocito (IPL) medio fue de 175,85 mil/mm^3^ (DE=90,86), presentando la mayoría (n=59; 72,84%) un IPL bajo. En cuanto a los biomarcadores, la Troponina I promedio fue de 3,23 pg/mL, advirtiéndose que solo 1 participante presentó índice elevado, mientras que para la Proteína C reactiva (PCR) el promedio fue de 6,83 mg/dL y 30 participantes presentaron alto riesgo cardiovascular.

**Tabla 2-:** Hemograma, INL*, IPL^†^, PCR^‡^y troponina de pacientes oncológicos remitidos a quimioterapia ambulatoria (n = 84). Vitória, ES, Brasil, 2022-2023

**Variables**	**n**	**%**
**Glóbulos rojos (millones/mm** ^3^ **)**		
Media (Desviación Estándar)	4,30 (0,56)	-
Mediana (Rango Intercuartílico)	4,27 (3,94 - 4,60)	-
**Hemoglobina (g/dL)**		
Media (Desviación Estándar)	12,53 (1,69)	-
Mediana (Rango Intercuartílico)	12,60 (11,60 - 13,50)	-
**Hematocrito (%)**		
Media (Desviación Estándar)	37,47 (4,67)	-
Mediana (Rango Intercuartílico)	37,40 (35,30 - 40,50)	-
**Leucocitos (millones/mm** ^3^ **)**		
Media (Desviación Estándar)	7,38 (2,57)	-
Mediana (Rango Intercuartílico)	6,95 (5,78 - 8,25)	-
**Plaquetas (millones/mm** ^3^ **)**		
Media (Desviación Estándar)	312,00 (108,47)	-
Mediana (Rango Intercuartílico)	294,00 (237,00 - 365,00)	-
INL* (millones/mm ^3^ **)**		
Media (Desviación Estándar)	2,91 (3,71)	-
Mediana (Rango Intercuartílico)	1,96 (1,57 - 2,66)	-
INL* (millones/mm ^3^ **)**		
Bajo	62	76,54
Alto	19	23,46
**IPL** ^†^ **(millones/mm** ^3^ **)**		
Media (Desviación Estándar)	175,81 (90,86)	-
Mediana (Rango Intercuartílico)	147,92 (115,90 - 202,31)	-
**IPL** ^†^ **(millones/mm** ^3^ **)**		
Bajo	59	72,84
Alto	22	27,16
**Troponina I (pg/mL)**		
Media (Desviación Estándar)	3,23 (5,44)	-
Mediana (Rango Intercuartílico)	1,50 (0,80 - 2,83)	-
**Troponina I (pg/mL)**		
Normal	83	98,81
Alto	01	1,19
**PCR** ^‡^ **(mg/dL)**		
Media (Desviación Estándar)	6,83 (18,84)	-
Mediana (Rango Intercuartílico)	1,67 (0,68 - 4,41)	-
**PCR** ^‡^ **(mg/dL) (riesgo cardiovascular)**		
Bajo	30	35,71
Moderado	24	28,57
Alto	30	35,71

*INL = Índice Neutrófilos/Linfocitos; ^†^IPL = Índice Plaquetas/Linfocitos; ^‡^PCR = Proteína C Reactiva

En la Tabla 3, respecto a la clase de quimioterapia, se observó que la combinación de agente taxano, agente alquilante, antraciclina y anticuerpo monoclonal fue la más frecuente entre pacientes sin prolongación del intervalo QTc (n=34) y también entre aquellos con QTc prolongado (n=4). La combinación de antimetabolito y derivado de platino se utilizó en 2 pacientes sin prolongación y en 1 con prolongación, mientras que la combinación de derivado de platino y agente taxano se utilizó en 11 pacientes sin prolongación y en 1 paciente con prolongación. Las otras combinaciones presentaron frecuencias más bajas. No hubo asociación estadísticamente significativa entre las diferentes combinaciones de quimioterapia y la ocurrencia de prolongación del intervalo QTc (p=0,285).

En cuanto a los niveles de PCR, se observó que la mayoría de los pacientes con QTc prolongado presentaban PCR elevada (n=6), mientras que entre los que no presentaban prolongación, la distribución fue más equilibrada entre las categorías: baja (n=25), moderada (n=20) y alta (n=23). Aun así, no se identificó una asociación estadísticamente significativa entre el riesgo cardiovascular estimado por el PCR y la presencia de prolongación del QTc (p=0,329).

En la Tabla 4, el análisis de la asociación entre el ECG y la PCR, así como las variables sociodemográficas y clínicas, reveló, en general, una falta de significación estadística para la mayoría de las variables estudiadas. Sin embargo, se observó una asociación significativa entre la circunferencia de la cintura y la FC (p=0,037), siendo la taquicardia más frecuente en pacientes con mayor riesgo de adiposidad abdominal, y también entre la presencia de ondas T invertidas y el IMC (p=0,005), siendo este hallazgo más común en personas con un IMC superior a 20,5, lo que podría sugerir una relación entre el sobrepeso/obesidad y las alteraciones de la repolarización ventricular.

La Tabla 5 muestra la asociación entre la historia clínica, el ECG y la PCR. Se observó una asociación estadísticamente significativa entre el tabaquismo y el ritmo cardíaco (p=0,010), con mayor frecuencia de ritmo irregular en fumadores. Los demás parámetros electrocardiográficos (FC, intervalo QTc, elevación del segmento ST, onda T invertida y QRS ensanchado) no mostraron una asociación significativa con las comorbilidades evaluadas (p>0,05). Respecto a la PCR, se observaron asociaciones significativas con la DM (p=0,033) y el consumo de alcohol (p=0,033), siendo los niveles elevados más frecuentes en personas con diabetes y consumidores de alcohol.

**Tabla 3-:** Asociación del intervalo QTc* prolongado con clase de quimioterapia y PCR^†^(riesgo cardiovascular) (n = 78). Vitória, ES, Brasil, 2022-2023

**Variables**	Intervalo QTc prolongado*		**p** ^‡^
	**No**	Sí	
**Clase de quimioterapia**			0,285
Antimetabolito + Derivado de Platino	2	1	
Derivado de Platino + Agente Taxano	11	1	
Agente Taxano + Agente Alquilante + Antraciclina + Anticuerpo Monoclonal	34	4	
Agente Taxano + Agente Alquilante + Derivado de Platino	1	1	
Antimetabolito + Inhibidor de Topoisomerasa + Derivado de Platino + Agente Taxano + Análogo de Ácido Fólico	20	3	
**PCR** ^†^ **(riesgo cardiovascular)**			0,329
Bajo	25	2	
Moderado	20	2	
Alto	23	6	

*QTc = QT corregido; ^†^PCR = Proteína C Reactiva; ^‡^Valor p calculado según la prueba exacta de Fisher

**Tabla 4-:** Asociación de los parámetros electrocardiográficos* de pacientes con cáncer remitidos a quimioterapia ambulatoria con PCR^†^ (riesgo cardiovascular) y variables sociodemográficas y clínicas (n = 78). Vitória, ES, Brasil, 2022-2023

**Variables**	**Ritmo**				**FC** ^‡^					**Intervalo QTc prolongado**				**Onda T invertida**					**QRS** ^||^ **ensanchado**		
	**Regular**	**Irregular**	p**		**Bradicardia**	**Normocardia (60-100)**	**Taquicardia**	p**		**No**	**Sí**	p**		**No**	**Sí**	**V1**	**Valor p**		**No**	**Sí**	p**
**PCR** ^†^ **(riesgo cardiovascular)**			0,837					0,600				0,329					0,454				0,378
Bajo	26	1			4	23	0			25	2			24	3	0			25	2	
Moderado	20	1			3	18	1			20	2			20	1	1			21	1	
Alto	28	1			3	23	3			23	6			28	1	0			29	0	
**Sexo**			0,066					0,302				0,134					0,727				0,190
Masculino	19	2			4	16	2			17	5			20	2	0			20	2	
Femenino	55	1			6	48	2			51	5			52	3	1			55	1	
**Rango de edad**			0,340					0,701				0,434					0,280				0,471
<50 años	19	1			1	18	1			18	2			20	0	0			20	0	
50-64 años	32	0			4	27	2			30	3			31	2	0			32	1	
≥65 años	23	2			5	19	1			20	5			21	3	0			23	2	
**Color de piel autoinformado**			1,000					0,884				0,697					0,395				1,000
Blanco	29	1			5	24	1			27	3			28	1	1			29	1	
Negro	9	0			2	7	0			7	2			8	1	0			9	0	
Marrón	28	2			3	25	3			27	4			29	2	0			29	2	
Amarillo	3	0			0	3	0			3	0			2	1	0			3	0	
Sin información	5	0			0	5	0			4	1			5	0	0			5	0	
**IMC** ^¶^			0,101					1,000				0,241					**0,005**				0,076
Por debajo de 20,5	1	1			0	2	0			1	1			0	2	0			1	1	
Por encima de 20,5	73	2			10	62	4			67	9			72	3	1			74	2	
**Circunferencia de la cintura**			0,290					**0,037**				0,590					0,712				0,763
Normal	13	2			0	15	0			14	1			13	2	0			14	1	
Riesgo elevado	20	0			6	14	0			16	4			19	1	0			20	0	
Riesgo muy elevado	41	1			4	35	4			38	5			40	2	1			41	2	
**Circunferencia abdominal**			0,387					0,062				1,000					0,796				0,571
Normal	30	2			7	26	0			29	4			30	3	0			31	2	
Obeso	44	1			3	38	4			39	6			42	2	1			44	1	
**Circunferencia abdominal**			1,000					0,755				0,699					0,151				0,145
Normal	18	1			2	17	0			16	3			16	3	0			17	2	
Obeso	56	2			8	47	4			52	7			56	2	1			58	1	
**Glucemia**			0,425					0,014				0,398					0,450				0,576
Normal	30	1			5	27	0			30	2			30	2	0			30	2	
Pre-diabético	13	2			2	11	2			13	2			12	2	1			14	1	
Diabético	24	0			0	22	2			19	5			23	1	0			24	0	
Sin información	7	0			3	4	0			6	1			7	0	0			7	0	
**Estadificación**			0,738					0,306				0,143					0,199				1,000
I	8	0			2	6	0			7	1			7	1	0			8	0	
II	42	1			6	36	2			37	7			43	1	0			42	2	
III	22	2			2	21	1			23	1			20	3	1			23	1	
Sin información	2	0			0	1	1			1	1			2	0	0			2	0	
**Clase de quimioterapia**			0,118					0,076				0,285					0,381				0,301
Antimetabolito + Derivado de Platino	2	0			2	1	0			2	1			3	0	0			3	0	
Derivado de Platino + Agente Taxano	12	0			3	8	1			11	1			10	2	0			11	1	
Agente Taxano + Agente Alquilante + Antraciclina + Anticuerpo Monoclonal	3	1			2	35	1			34	4			37	1	0			38	0	
Agente de Taxano + Agente Alquilante + Derivado de Platino	2	0			0	2	0			1	1			2	0	0			2	0	
Antimetabolito + Inhibidor de Topoisomerasa + Derivado de Platino + Agente Taxano + Análogo de Ácido Fólico	21	2			3	18	2			20	3			20	2	1			21	2	

*ECG = Electrocardiograma; ^†^PCR = Proteína C reactiva; ^‡^FC = Frecuencia cardíaca;^§^QTc = Intervalo QT corregido; ^||^QRS = Complejo QRS; ^¶^IMC = Índice de Masa Corporal; **p = Valor p calculado con base en la prueba exacta de Fisher

**Tabla 5 -:** Asociación entre los antecedentes de pacientes con cáncer con indicación de quimioterapia ambulatoria y ECG* y Proteína C reactiva (riesgo cardiovascular) (n = 78). Vitória, ES, Brasil, 2022-2023

**Variables**		**HAS** ^†^				**IAM** ^‡^				**Dislipidemia**				**Diabetes mellitus**				**ACV** ^§^				**Enfermedad valvular**				**Consume tabaco**				**Consume bebidas alcohólicas**				**Antecedentes familiares de cardiopatía**		
		**No**	**Sí**	**p** ^||^		**No**	**Sí**	**p** ^||^		**No**	**Sí**	**p** ^||^		**No**	**Sí**	**p** ^||^		**No**	**Sí**	**p** ^||^		**No**	**Sí**	**p** ^||^		**No**	**Sí**	**p** ^||^		**No**	**Sí**	**p** ^||^		**No**	**Sí**	**p** ^||^
**Ritmo**				0,793				1,000				1,000				0,582				1,000				1,000				**0,010**				1,000				1,000
	Regular	39	35			70	4			65	9			60	14			71	3			73	1			70	3			63	10			25	48	
	Irregular	1	2			3	0			3	0			2	1			3	0			3	0			1	1			2	0			1	2	
** **	Sin información	1	0			1	0			1	0			1	0			1	0			1	0			0	1			1	0			0	1	
**FC** ^¶^				0,343				1,000				1,000				0,328				1,000				1,000				0,690				0,628				0,188
	Bradicardia	7	3			10	0			9	1			8	2			10	0			10	0			9	1			9	1			1	9	
	Normocardia (60-100)	33	31			60	4			56	8			53	11			61	3			63	1			58	4			54	8			23	40	
** **	Taquicardia	1	3			4	0			4	0			2	2			4	0			4	0			4	0			3	1			2	2	
**Intervalo QTc prolongado** **				0,504				0,429				0,324				0,093				0,341				1,000				0,477				0,337				0,151
	No	37	31			65	3			61	7			57	11			66	2			67	1			63	4			59	8			25	42	
** **	Sí	4	6			9	1			8	2			6	4			9	1			10	0			8	1			7	2			1	9	
**Elevación ST** ^††^				0,474				1,000				1,000				1,000				1,000				1,000				1,000				1,000				1,000
	No	41	36			73	4			68	9			62	15			74	3			76	1			70	5			65	10			26	50	
** **	Sí	0	1			1	0			1	0			1	0			1	0			1	0			1	0			1	0			0	1	
**Onda T invertida**				0,114				1,000				0,533				0,658				0,216				1,000				1,000				1,000				0,214
	No	40	32			68	4			64	8			57	15			70	2			71	1			66	5			61	10			26	45	
	Sí	1	4			5	0			4	1			5	0			4	1			5	0			4	0			4	0			0	5	
** **	Versión 1	0	1			1	0			1	0			1	0			1	0			1	0			1	0			1	0			0	1	
**QRS ensanchado** ^‡‡^				0,601				0,148				1,000				1,000				0,112				1,000				1,000				1,000				0,547
	No	40	35			72	3			66	9			60	15			73	2			74	1			69	5			64	10			26	48	
	Sí	1	2			2	1			3	0			3	0			2	1			3	0			2	0			2	0			0	3	
**Proteína C Reactiva (riesgo cardiovascular)**				0,247				0,674				0,741				**0,033**				0,774				1,000				0,431				**0,033**				0,688
	Bajo	13	14			26	1			25	2			25	2			26	1			27	0			24	1			25	0			9	18	
	Moderado	15	7			20	2			19	3			19	3			22	0			22	0			19	3			18	4			6	16	
** **	Alto	13	16			28	1			25	4			19	10			27	2			28	1			28	1			23	6			11	17	

*ECG = Electrocardiograma; ^†^HAS = Hipertensión Arterial Sistémica; ^‡^IAM = Infarto Agudo de Miocardio; ^§^ACV = Accidente Cerebrovascular; ^||^p = Valor p calculado con base en la prueba exacta de Fisher; ^¶^FC = Frecuencia Cardíaca; **QTc = QT corregido; ^††^ST = Segmento ST; ^‡‡^QRS = Complejo QRS

## Discusión

En el presente estudio, la mayoría de los participantes eran mujeres, con mayor incidencia de diagnóstico de cáncer de mama, seguido por cáncer de colon, bronquios y pulmón. Desde esta perspectiva, la evidencia corrobora que el cáncer de mama es el tumor que más afecta a las mujeres a nivel mundial, cuando se excluye el cáncer de piel no melanoma^(31)^. Sin embargo, esta distribución difiere de las estimaciones internacionales^(32)^ y nacionales^(33)^, lo que puede estar asociado a factores socioeconómicos y ambientales, y otras cuestiones relacionadas con el perfil de la población de estudio. En vista de esto, un estudio demuestra que los casos de neoplasias malignas de mama han aumentado progresivamente con el avance de la industrialización y la urbanización^(34)^.

El promedio de edad (57-68 años) coincide con el perfil de edad comúnmente descripto para pacientes con cáncer^(33-34)^. El envejecimiento, en sí mismo, es un factor de riesgo de carcinogénesis, al estar asociado con comorbilidades e inestabilidad genómica, como la mutación de la telomerasa. Por lo tanto, la edad biológica se relaciona con un mayor riesgo de cualquier tipo de cáncer^(35)^.

En lo que se refiere a la terapia, se observó la prevalencia del régimen de quimioterapia compuesto por taxanos, agentes alquilantes, antraciclinas y anticuerpos monoclonales, comúnmente recomendado para el tratamiento de tumores sólidos, como el cáncer de mama, vejiga y pulmón. Estas clases de agentes quimioterapéuticos se reconocen como algunos de los principales agentes con alto potencial cardiotóxico^(7,36)^.

Un estudio de pacientes con cáncer de mama en etapa temprana demuestra que los efectos cardiotóxicos de la doxorrubicina (DOX) pueden aparecer tardíamente, años después de la exposición a la quimioterapia, lo que destaca la importancia de la identificación temprana de las personas en riesgo^(7,37)^. Además de las antraciclinas, el Docetaxel y la Ciclofosfamida también tienen efectos tóxicos sobre el miocardio, aunque sus mecanismos aún no se comprenden por completo. Ambos se asocian con el riesgo de disfunción ventricular izquierda (DVI)^(38-40)^, que representa la principal manifestación cardiotóxica^(41)^.

En el estudio, el 23,46% (n=19) y el 27,16% (n=22) de las pacientes presentaron valores basales elevados de INL y IPL, respectivamente. Por el contrario, un estudio de pacientes con diagnóstico de cáncer de mama en tratamiento con antraciclinas no identificó cambios en los valores basales de INL; sin embargo, se observó un aumento de este marcador tras la exposición a la quimioterapia, asociado con un riesgo cuatro veces mayor de desarrollar disfunción cardíaca, lo que sugiere una mayor influencia de la antraciclina que la inflamación basal causada por la enfermedad^(40)^.

Otro estudio, que relacionó el INL y el IPL con el estado nutricional, los factores clínicos y sociodemográficos, y la calidad de vida de mujeres hospitalizadas con cáncer de mama no metastásico, halló que existe un alto riesgo en personas con valores medios y altos de INL e IPL, lo que implica una baja actividad antitumoral y un peor pronóstico^(14)^. Estos biomarcadores, relacionados con la inflamación sistémica, se han estudiado como herramientas accesibles de estratificación del riesgo^(41)^.

La PCR, también considerada un importante marcador inflamatorio, fue elevada en el 35,71% de los pacientes (n=30), lo que indica un mayor riesgo cardiovascular. Además, una revisión sistemática mostró que la PCR, junto con la troponina y los péptidos natriuréticos tipo B (BNP), se relaciona con resultados cardiovasculares adversos graves, como infarto de miocardio e insuficiencia cardíaca^(42)^. En este estudio, se observó una asociación entre DM y PCR, lo que indica una mayor presencia de un proceso inflamatorio en los pacientes. Esta condición contribuye al aumento del riesgo cardiovascular, así como a la progresión de las complicaciones vasculares^(43)^.

El monitoreo de la cardiotoxicidad y la inflamación sistémica a través de la medición de biomarcadores séricos, como INL, IPL, Troponina I y PCR, es un método accesible, mínimamente invasivo y de bajo costo que contribuye a la estratificación del riesgo y la detección temprana de esta condición antes de la terapia inicial, además de contribuir al diagnóstico temprano de ECV tanto durante como después del tratamiento, brindando apoyo para detectar pacientes con cáncer que pueden beneficiarse de tratamientos cardioprotectores o que requieren seguimiento a largo plazo^(44-46)^.

Múltiples factores de riesgo cardiovascular preexistentes, como hipertensión, diabetes mellitus, dislipidemia y obesidad, son ampliamente reconocidos en la literatura como afecciones que aumentan el riesgo de toxicidad cardiovascular asociada con el tratamiento oncológico^(36)^. Sin embargo, en la presente muestra, no se encontraron asociaciones estadísticamente significativas entre las variables clínicas y de laboratorio y la mayoría de los parámetros electrocardiográficos evaluados. A pesar de esto, se destaca la identificación de una relación entre el IMC y la onda T invertida (p=0,005). Este hallazgo es consistente con estudios que informan el impacto del IMC alto en la función eléctrica miocárdica, favoreciendo cambios en la repolarización^(47-48)^.

Aunque la prolongación del intervalo QTc no se asoció significativamente con las variables investigadas, se observó que 10 pacientes ya presentaban esta alteración al inicio, lo que refuerza la necesidad de monitorización continua. La evidencia muestra que la prolongación del intervalo QTc puede exacerbarse durante el tratamiento, especialmente con el uso acumulativo de antraciclinas y taxanos, lo que aumenta el riesgo de arritmias graves^(4,49)^.

La fibrilación auricular, las palpitaciones ventriculares ectópicas y la prolongación del intervalo QTc se encuentran entre las arritmias más comunes en pacientes oncológicos y pueden agravarse durante el tratamiento^(50-52)^. En este contexto, la evaluación electrocardiográfica debe incorporarse sistemáticamente desde la fase inicial del tratamiento.

Es importante considerar que la ausencia de asociaciones observadas en este estudio podría estar relacionada con que los pacientes se encontraban en la fase pre-quimioterapia, un período en el que los efectos cardiotóxicos asociados al tratamiento aún no se habían manifestado. Por lo tanto, si bien no se observaron asociaciones significativas al inicio, es importante destacar que estos factores están bien establecidos en la literatura como predictores de un mayor riesgo de desarrollar cardiotoxicidad, especialmente en pacientes expuestos a agentes como las antraciclinas^(7,36)^.

De hecho, estudios previos muestran que los individuos con estas comorbilidades tienen hasta 12 veces más probabilidades de desarrollar disfunción cardíaca inducida por antraciclinas^(53)^. Además, según las Guías de Práctica Clínica de la Sociedad Americana de Oncología Clínica, la presencia de múltiples factores de riesgo cardiovascular, asociados al uso de antraciclinas y la edad mayor de 60 años, constituye un escenario de alto riesgo para el desarrollo de disfunción cardíaca^(54-55)^. Por lo tanto, este análisis confirma aún más el impacto de las comorbilidades en el riesgo cardiotóxico y destaca la importancia del control de las enfermedades subyacentes, además de la evaluación y el seguimiento de los pacientes por un equipo multidisciplinario centrado en el sistema cardiovascular desde el inicio hasta el tratamiento posterior a la quimioterapia como una forma de prevención, siguiendo las recomendaciones de la Guía Brasileña de Cardio-Oncología^(36)^.

También es importante explorar el potencial del ECG y de los biomarcadores séricos, como el INL, el IPL, la troponina I y la PCR, como herramientas de diagnóstico y seguimiento de las alteraciones cardíacas, así como para la estratificación del riesgo cardiovascular, con el objetivo de identificar precozmente la cardiotoxicidad y seleccionar pacientes que puedan beneficiarse de intervenciones cardioprotectoras^(41)^.

Aunque los biomarcadores inflamatorios y cardíacos analizados en este estudio tienen reconocida relevancia clínica, es importante considerar que la evaluación se realizó en la fase pre-quimioterapia, un período anterior a la manifestación completa de los efectos citotóxicos e inflamatorios más intensos resultantes del tratamiento antineoplásico. Esto puede explicar la falta de asociaciones estadísticamente significativas entre estos marcadores y los parámetros clínicos o electrocardiográficos evaluados. La baja elevación de troponina I, observada en un solo paciente, limita su aplicabilidad como marcador basal, aunque su utilidad pronóstica está bien establecida en fases posteriores del tratamiento, especialmente como predictor de disfunción ventricular inducida por antraciclinas^(8,28)^. De manera similar, la PCR elevada, identificada en el 35,7% de la muestra, cuando se utiliza de forma aislada, presenta una baja especificidad como marcador de riesgo cardiovascular y puede estar influenciada por otras condiciones inflamatorias, infecciosas o metabólicas, como el estado nutricional, la presencia de diabetes mellitus o la carga tumoral^(15,42)^. Sin embargo, la detección temprana de estos biomarcadores, incluso a niveles subclínicos, puede contribuir a la estratificación individualizada del riesgo y orientar las estrategias preventivas en cardio-oncología, especialmente en pacientes con comorbilidades o factores de riesgo modificables. Este enfoque está respaldado por las guías internacionales y la evidencia más reciente sobre biomarcadores en la monitorización de la cardiotoxicidad^(41,54)^.

Los profesionales sanitarios, especialmente el personal de enfermería, desempeñan un papel fundamental en la vigilancia clínica de los pacientes oncológicos. Es fundamental que monitoricen y evalúen los biomarcadores séricos y los ECG para asegurar la identificación temprana y la estratificación del riesgo cardiovascular, tanto durante el tratamiento como en el seguimiento (*follow-up*)*.* Dada la cardiotoxicidad reconocida de varios agentes quimioterapéuticos, la intervención oportuna puede minimizar las complicaciones y mejorar el pronóstico^(7,36)^.

En ese contexto, se destaca la creciente incorporación de las ciencias ómicas a la práctica clínica de Enfermería^(56-58)^, lo que refuerza la importancia de la Enfermería de Precisión^(51-52)^. La aplicación de estrategias individualizadas, basadas en biomarcadores específicos y las características individuales de cada paciente, contribuye a una atención más eficaz y personalizada, centrada en el individuo y orientada a la toma de decisiones clínicas basadas en la evidencia^(58-60)^.

Por lo tanto, es esencial adoptar protocolos de seguimiento, combinados con intervenciones terapéuticas basadas en la evidencia, con el objetivo de mitigar la cardiotoxicidad, reducir la morbilidad y la mortalidad cardiovascular y, en consecuencia, mejorar el pronóstico de los pacientes oncológicos^(60)^. Las directrices internacionales como las de la *European Society of Cardiology* o la *European Society for Medical Oncology*(ESMO), así como las de la *American Society of Clinical Oncology* (ASCO), refuerzan la necesidad de una monitorización estructurada y sistemática de los pacientes expuestos a agentes cardiotóxicos. La adopción de estas recomendaciones permite la detección temprana de la disfunción miocárdica subclínica, lo que permite intervenciones oportunas y eficaces para prevenir la progresión a insuficiencia cardíaca y otros eventos cardiovasculares adversos^(7)^.

Los hallazgos de este estudio tienen implicaciones relevantes para la práctica clínica de enfermería, especialmente en el contexto de la enfermería oncológica. La identificación de biomarcadores inflamatorios y cardíacos en la fase previa a la quimioterapia, incluso a niveles subclínicos, resalta la necesidad de una acción proactiva del equipo de enfermería en la estratificación temprana del riesgo cardiovascular^(60-61)^. Marcadores como INL, IPL, troponina I y PCR, por ser accesibles y de bajo costo, pueden incorporarse como herramientas complementarias a la anamnesis y al examen físico en el cribado cardiovascular, apoyando la planificación de intervenciones personalizadas y la vigilancia continua durante todo el tratamiento oncológico. La realización sistemática de pruebas como el ECG, combinada con la interpretación crítica de los biomarcadores, fortalece el papel de la Enfermería en la detección temprana de alteraciones cardiovasculares, la comunicación con el equipo multidisciplinario y la adopción de estrategias cardioprotectoras.

Además, la integración de estas prácticas en el protocolo de evaluación inicial del paciente oncológico promueve una atención integral, segura y basada en evidencia, en consonancia con los principios de la Enfermería de Precisión y las guías nacionales e internacionales de cardio-oncología. Esta evidencia refuerza la importancia de la Enfermería como protagonista en la detección, el seguimiento y la educación en salud cardiovascular en la atención oncológica, contribuyendo directamente a la prevención de complicaciones y a la mejora de los resultados clínicos de estos pacientes.

Las limitaciones de este estudio incluyen: I. La ligera elevación de Troponina I, detectada en un solo paciente, que limita la interpretación de la aplicabilidad de este marcador en condiciones basales; II. El estudio se realizó en un solo centro especializado en el tratamiento del cáncer, con una muestra relativamente pequeña y heterogénea, lo que puede comprometer la validez externa y la generalización de los hallazgos a otras poblaciones o entornos clínicos; III. Aunque el número total de pacientes alcanzó la muestra mínima estimada (n=84), algunos análisis específicos presentaron pérdidas puntuales en la muestra debido a la inconsistencia o ausencia de datos de laboratorio y/o electrocardiográficos, lo que redujo el número de participantes en determinadas variables y pudo haber afectado el poder estadístico de dichos análisis; IV. La ausencia de un grupo control compuesto por pacientes con cáncer que no se sometieran a quimioterapia o sin factores de riesgo cardiovascular; V. El diseño transversal del estudio, que impide el seguimiento longitudinal de los cambios inflamatorios y electrocardiográficos a lo largo del tratamiento oncológico; VI. La falta de realización de pruebas complementarias importantes, como la medición de NT- ProBNP y la ecocardiografía, que podrían contribuir a una evaluación más completa de la función cardíaca; VII. El uso de la PCR como marcador aislado en la estratificación del riesgo cardiovascular, lo que limita su aplicabilidad clínica, especialmente en ausencia de otros parámetros complementarios.

No obstante, a pesar de sus limitaciones, nuestro estudio presenta aspectos relevantes, como: I. Evaluación de medidas subjetivas y objetivas (mediante la medición de biomarcadores); II. Recopilación estandarizada de biomarcadores y datos clínicos antes de la infusión de la primera sesión de quimioterapia, lo que garantiza la fiabilidad y validez de los datos; III. Relevancia clínica de los resultados para la estratificación temprana del riesgo cardiovascular, con el objetivo de mejorar el bienestar y el pronóstico de los pacientes oncológicos sometidos a quimioterapia. Sin embargo, destacamos que los datos presentados ofrecen evidencia preliminar importante sobre la evaluación cardiovascular basal en pacientes con cáncer en la fase pre-quimioterapia, lo que contribuye a la base de futuras investigaciones multicéntricas y longitudinales.

Se recomienda la realización de estudios longitudinales con una muestra representativa y bien diseñada para comprender las tendencias en los diagnósticos de cáncer a lo largo del tiempo e identificar los factores de riesgo cardíaco asociados durante el *follow-up*. Además, se sugiere investigar estrategias de prevención e intervención para mitigar el riesgo de daño cardíaco derivado de la quimioterapia, con especial atención a la cardio-oncología y la Enfermería de Precisión.

## Conclusión

En el presente estudio, la falta de asociaciones estadísticamente significativas entre la mayoría de los parámetros evaluados podría deberse a que los pacientes se encontraban en la fase pre-quimioterapia, período previo a la aparición de los efectos cardiotóxicos esperados. Sin embargo, se identificaron factores de riesgo cardiovascular relevantes, como la inflamación sistémica y el IMC elevado, que, junto con ciertos protocolos de quimioterapia, pueden aumentar el riesgo de disfunción cardíaca.

Estos hallazgos refuerzan la necesidad de incorporar rutinas sistemáticas de cribado cardiovascular antes de la primera infusión de quimioterapia, por tanto, desde el inicio del seguimiento oncológico, como estrategia esencial para la detección temprana de alteraciones cardiovasculares y para la implementación de intervenciones preventivas, personalizadas y oportunas.

## Data Availability

Todos los datos generados o analizados durante este estudio están incluidos en este artículo publicado.
